# Supplementary material for: Excitatory synapses and gap junctions cooperate to improve Pv neuronal burst firing and cortical social cognition in Shank2-mutant mice
Source: Nat Commun. 2021 Aug 25;12:5116. doi: 10.1038/s41467-021-25356-2 (PMC8387434; doi:10.1038/s41467-021-25356-2)
Supplement: Supplementary file 1 — Supplementary Information [file 41467_2021_25356_MOESM1_ESM.pdf]

Supplementary Information

Excitatory synapses and gap junctions cooperate to improve Pv neuronal burst firing and cortical social cognition in *Shank2*-mutant mice

Eunee Lee,[1,2,*] Seungjoon Lee,[3,*] Jae Jin Shin,[4,5,*] Woochul Choi,[6,*] Changuk Chung,[1] Suho Lee,[3] Jihye Kim,[1] Seungmin Ha,[1] Ryunhee Kim,[1] Taesun Yoo,[1] Ye-Eun Yoo,[3] Jisoo Kim,[3] Young Woo Noh,[3] Issac Rhim,[1] Soo Yeon Lee,[3] Woohyun Kim,[3] Taekyung Lee,[1] Hyogeun Shin,[5] Il-Joo Cho,[7] Karl Deisseroth,[8] Sang Jeong Kim,[4] Joo Min Park,[5,#] Min Whan Jung,[1,3,#] Se-Bum Paik,[6,#] and Eunjoon Kim[1,3,#]

[1]Center for Synaptic Brain Dysfunctions, Institute for Basic Science (IBS), Daejeon 34141, Korea; [2]Dept. of Anatomy, College of Medicine, Yonsei University, Seoul; [3]Department of Biological Sciences, KAIST, Daejeon 34141, Korea; [4]Department of Brain and Cognitive Science, College of Natural Science, Seoul National University, Seoul 08826, Korea; [5]Center for Cognition and Sociality, Institute for Basic Science (IBS), Daejeon, Korea; [6]Program of Brain and Cognitive Engineering, Department of Bio and Brain Engineering, Korea Advanced Institute for Science and Technology (KAIST), Daejeon 34141, Korea; [7]Center for BioMicrosystems, Brain Science Institute, Korea Institute of Science and Technology (KIST), Seoul, Korea; [8]Department of Bioengineering, Department of Psychiatry and Behavioral Sciences, Howard Hughes Medical Institute, Stanford University, Stanford, CA 94305, USA; *These authors contributed equally to the work; [#]Corresponding authors.

Supplementary figure legends

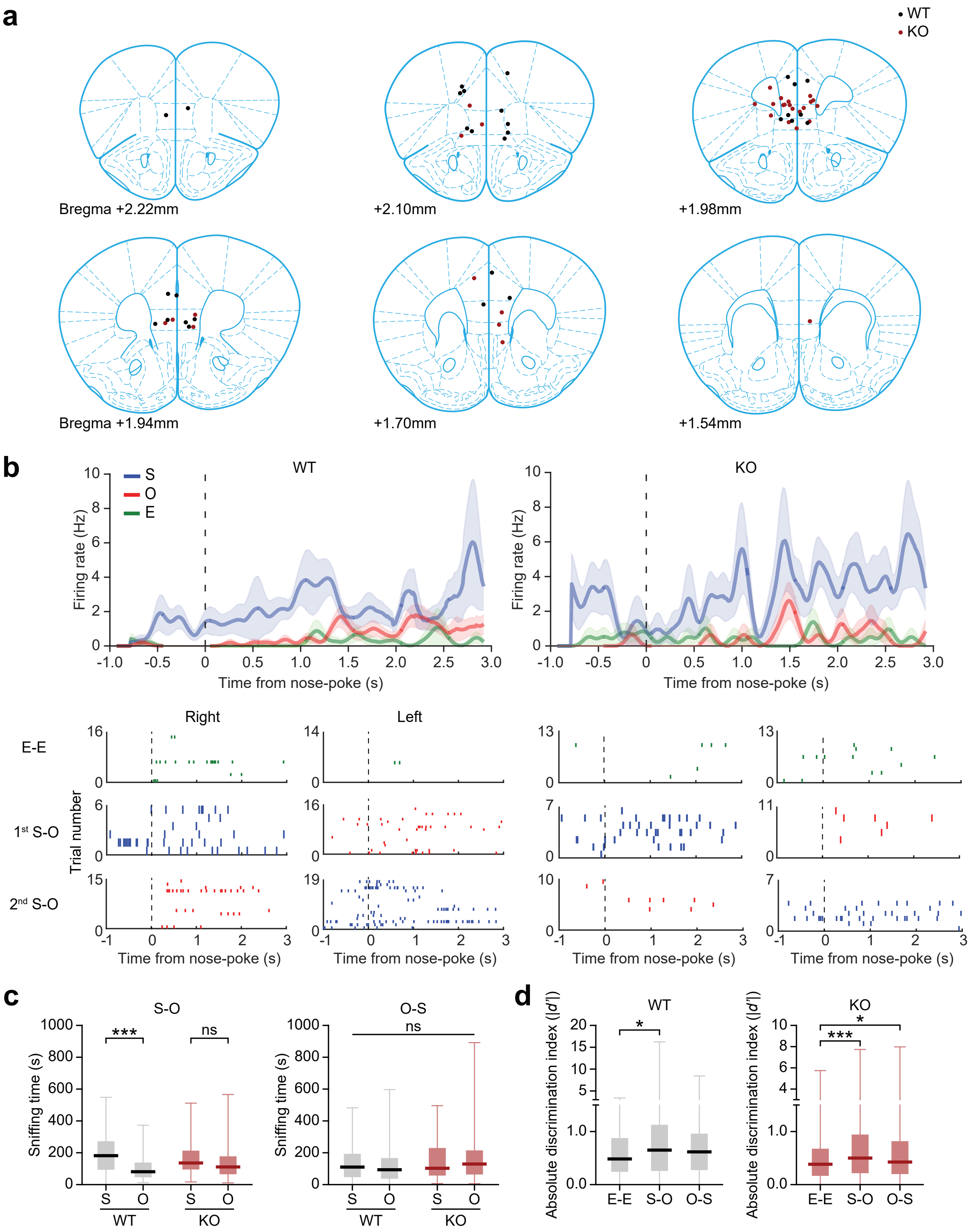

a
WT
KO
Bregma +2.22mm
+2.10mm
+1.98mm
Bregma +1.94mm
+1.70mm
+1.54mm
b
WT
KO
S
O
E
Firing rate (Hz)
Time from nose-poke (s)
Right
Left
E-E
1st S-O
2nd S-O
Trial number
Time from nose-poke (s)
c
S-O
O-S
Sniffing time (s)
***
ns
S
O
WT
KO
d
WT
KO
Absolute discrimination index (|d'|)
*
***
E-E
S-O
O-S

Supplementary figure 1. Positions of single-unit tetrode tips and examples of WT and *Shank2*$^{-/-}$ mPFC neurons that show target-specific or broadly-tuned neuronal firings.
(a) Positions of single-unit tetrode tips in mPFC regions.
(b) Examples of neurons in the mPFC of WT and *Shank2*$^{-/-}$ mice that show target-specific or broadly-tuned discharges. S, social; O, object; E, empty.
(c) Social and object target explorations in the first and second S-O session in the linear chamber, as shown by nose-poke time. Data: minimal, maximal, median, 25%, and 75% values. Data: mean ± SEM. (n = 56 experiments with 5 mice [WT] and 60, 5 [KO], ***p < 0.001, ns, not significant, two-way repeated-measures ANOVA).
(d) Discriminative neuronal responses for target pairs in the E-E, first S-O, and second S-O sessions, as shown by the discrimination index (*d'*; absolute values without consideration of response directions) [1,2]. Data: minimal, maximal, median, 25%, and 75% values. Data: mean ± SEM. (n = 293 neurons from 5 mice [WT] and 386, 5 [KO], *p < 0.05, ***p < 0.001, Friedman test with Dunn's multiple comparison).

See Source Data for raw data values and Supplementary Table 1 for statistical details.

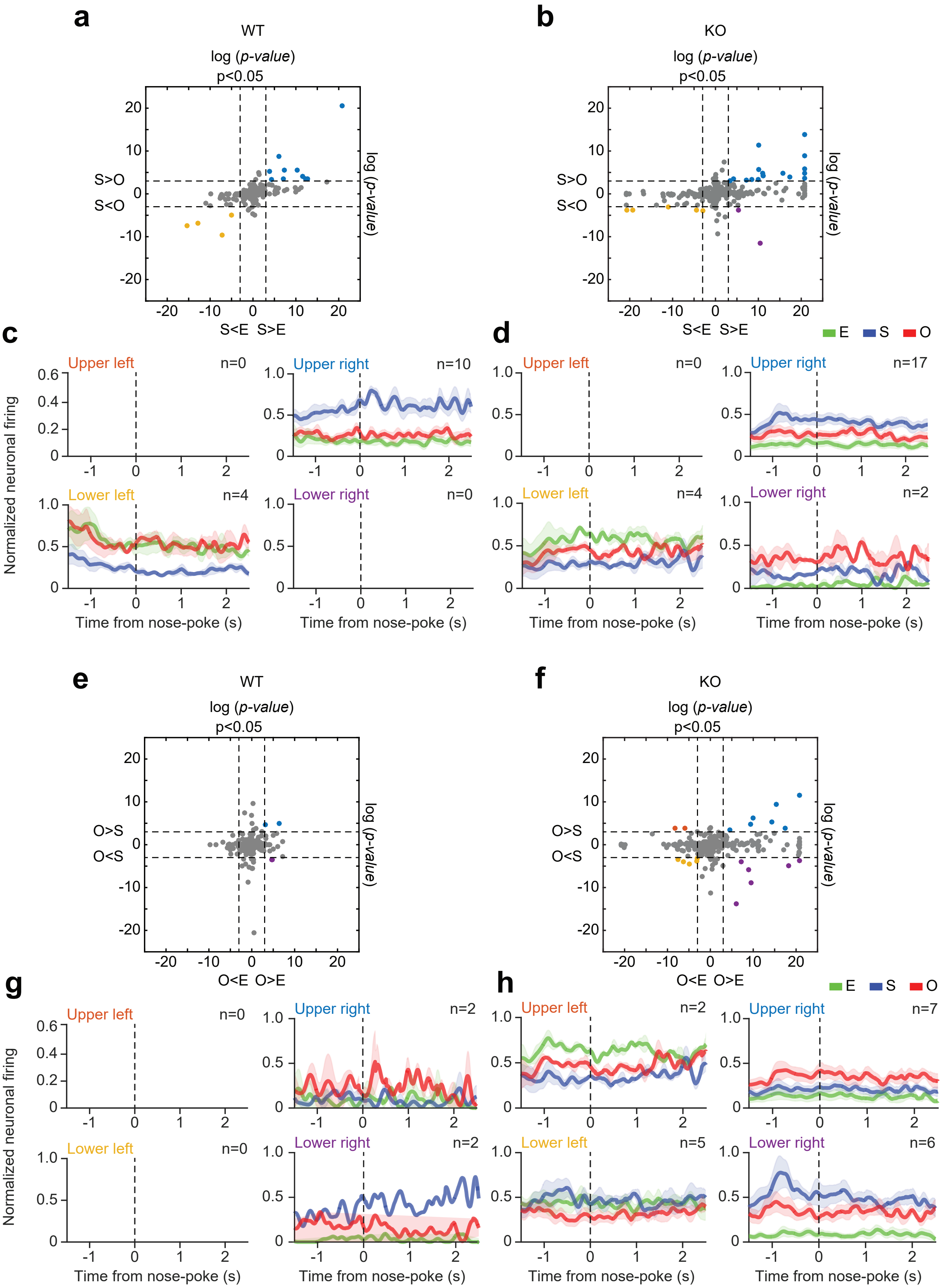
a
WT
log (p-value)
p<0.05
S>O
S<O
S<E S>E
log (p-value)
b
KO
log (p-value)
p<0.05
S>O
S<O
S<E S>E
log (p-value)
c
Upper left n=0
Upper right n=10
Lower left n=4
Lower right n=0
Normalized neuronal firing
Time from nose-poke (s)
d
E S O
Upper left n=0
Upper right n=17
Lower left n=4
Lower right n=2
Time from nose-poke (s)
e
WT
log (p-value)
p<0.05
O>S
O<S
O<E O>E
log (p-value)
f
KO
log (p-value)
p<0.05
O>S
O<S
O<E O>E
log (p-value)
g
Upper left n=0
Upper right n=2
Lower left n=0
Lower right n=2
Normalized neuronal firing
Time from nose-poke (s)
h
E S O
Upper left n=2
Upper right n=7
Lower left n=5
Lower right n=6
Time from nose-poke (s)

Supplementary figure 2. Discharge patterns of WT and *Shank2*$^{-/-}$ mPFC neurons grouped according to their discriminative discharges to S-O and S-E, or O-S and O-E, target contexts.
(a–d) Two-dimensional plots of p-values for the discrimination of S-O and S-E pairs of target contexts (or target pairs, in short) by total neurons (a and b; n = 293 and 429 neurons from WT and KO, respectively). Data: mean ± SEM. P-values for discriminative discharges for specific target pairs were determined by two-way ANOVA, as described previously (Lee et al., 2016). Dotted lines in panels a and b indicate $p = 0.05$ (two-way ANOVA with Tukey's test). Panels c and d indicate averaged discharges of all neurons that significantly ($p < 0.05$) discriminated specific target pairs plotted against the time window encompassing nose poke (number of neurons are indicated). Shading indicates standard error of the mean (SEM).

(e–h) Two-dimensional plots of p-values for the discrimination of O-S and O-E target pairs by total neurons (a and b; n = 293 and 439 neurons from WT and KO, respectively). Data: mean ± SEM. Averaged discharges of all neurons that significantly ($p < 0.05$; two-way ANOVA with Tukey's test) discriminated specific target sets plotted against the time window encompassing nose poke (c and d; numbers of neurons are indicated).

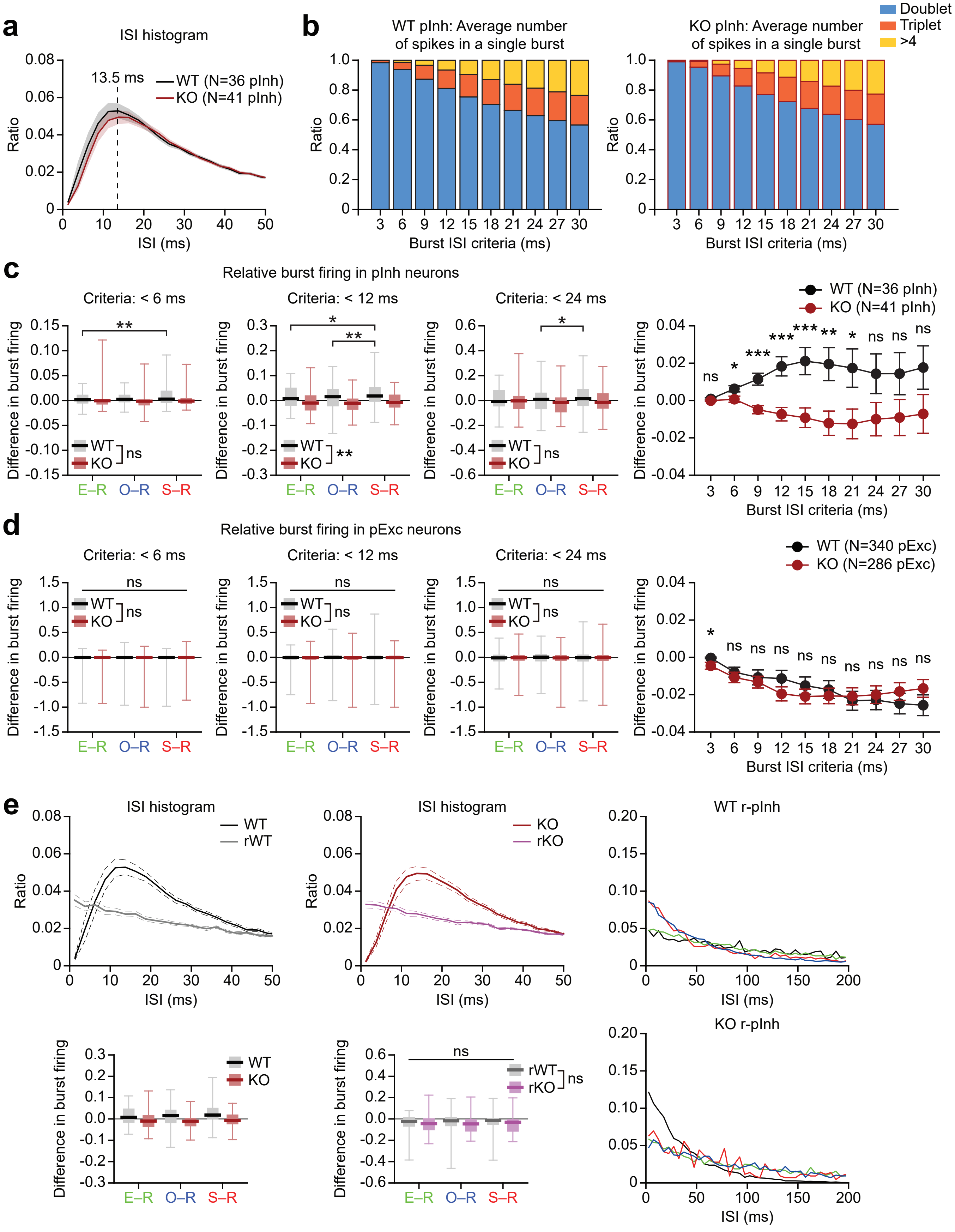
a
ISI histogram
13.5 ms
WT (N=36 pInh)
KO (N=41 pInh)
Ratio
ISI (ms)
b
WT pInh: Average number of spikes in a single burst
KO pInh: Average number of spikes in a single burst
Doublet
Triplet
>4
Ratio
Burst ISI criteria (ms)
c
Relative burst firing in pInh neurons
Criteria: < 6 ms
Criteria: < 12 ms
Criteria: < 24 ms
WT (N=36 pInh)
KO (N=41 pInh)
Difference in burst firing
WT
KO
ns
**
*
***
E–R
O–R
S–R
Burst ISI criteria (ms)
d
Relative burst firing in pExc neurons
Criteria: < 6 ms
Criteria: < 12 ms
Criteria: < 24 ms
WT (N=340 pExc)
KO (N=286 pExc)
Difference in burst firing
WT
KO
ns
*
E–R
O–R
S–R
Burst ISI criteria (ms)
e
ISI histogram
WT
rWT
KO
rKO
Ratio
ISI (ms)
WT r-pInh
KO r-pInh
Difference in burst firing
WT
KO
rWT
rKO
ns
E–R
O–R
S–R

Supplementary figure 3. Burst firing profile of WT and *Shank2*$^{-/-}$ pInh neurons.
(a) ISI histograms of WT and KO (*Shank2*$^{-/-}$) pInh neurons (black: WT, red: KO). Note that the two distributions of ISIs peak at 13.5 ms, forming the basis for the definition of burst firing as consecutive spikes with ISI < 12 ms.
(b) Number of spikes in each burst using various thresholds of burst definition. Note that as the ISI threshold of a burst increases, the number of spikes in each burst increases and that large portions (~60–80%) of the bursts across different thresholds, including 12 ms, contain two consecutive spikes.
(c) Bar graphs for Δburst firing (changes in high-frequency burst firings during S/O/E target encounter relative to the R state) for E, O, and S in WT and KO pInh neurons, with different burst thresholds. Note that levels of Δburst firing are always higher in WT than KO pInh neurons with three different burst thresholds (ISI < 6 ms, 12 ms, and 24 ms are shown as examples). Note also that the average difference in Δburst firing between WT and KO were significant in the ISI threshold range of 6-21 ms, as shown by the genotype differences at each ISI thresholds (graph on the right). Data: minimal, maximal, median, 25%, and 75% values. Data: mean ± SEM. (n = 36 neurons [WT] and 41 [KO], two-way ANOVA with Tukey's test [left three graphs]).
(d) Same as C, bur for pExc neurons. Note that different ISI thresholds for bursts did not lead to significant differences in Δburst firing between KO and WT pExc neurons. Data: minimal, maximal, median, 25%, and 75% values. Data: mean ± SEM. (n = 340 neurons [WT] and 286 [KO], two-way ANOVA with Tukey's test [left three graphs]).
(e) Shuffling spike times eliminates Δburst firing differences across targets in pInh neurons. rWT/KO, random shuffling in WT/KO. Data: minimal, maximal, median, 25%, and 75% values. Data: mean ± SEM. (n = 340 neurons [WT] and 286 [KO], two-way ANOVA with Tukey's test).

See Source Data for raw data values and Supplementary Table 1 for statistical details.

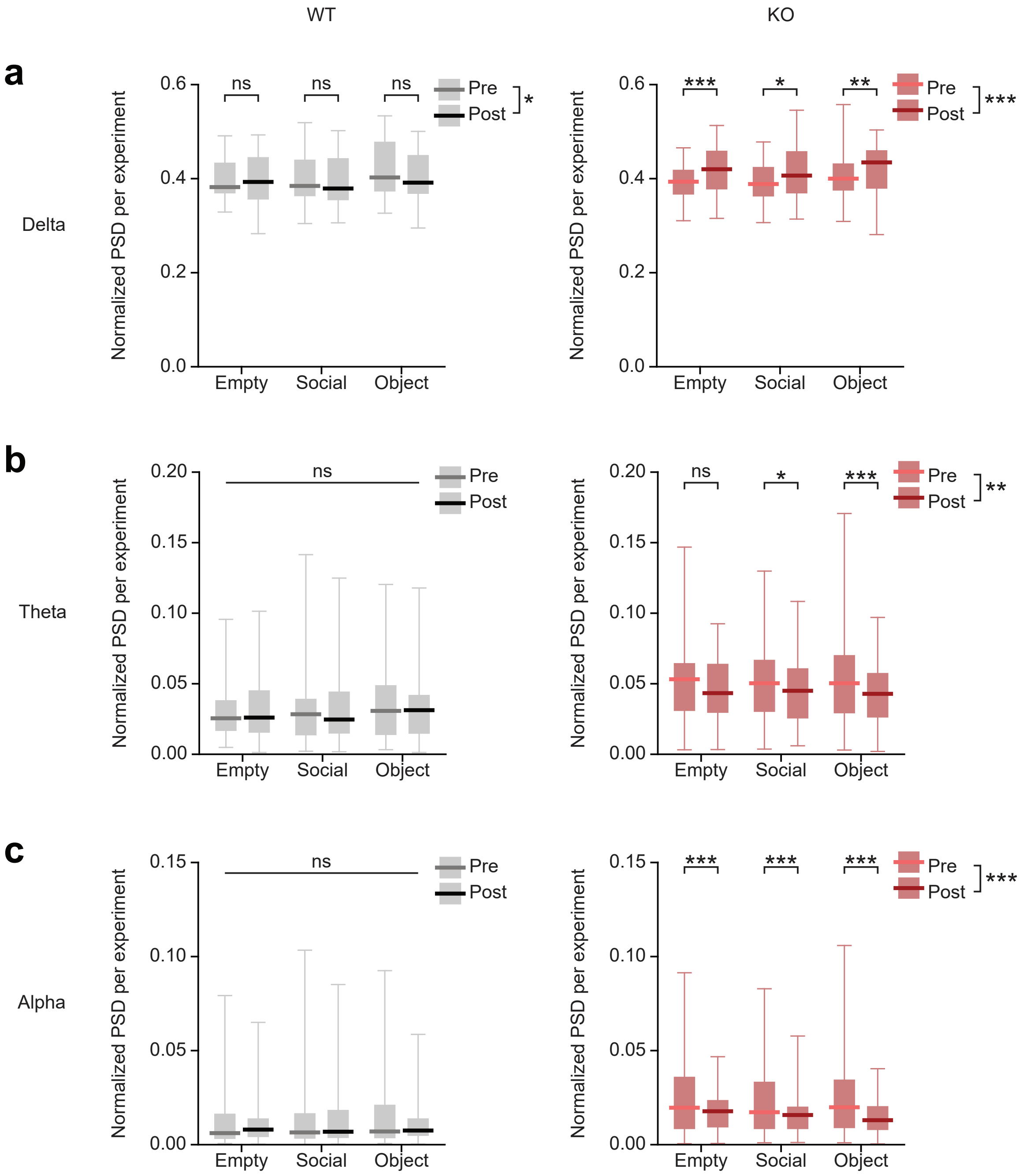

Supplementary figure 4. Changes in the spectral powers of target-induced oscillations in delta, theta, and alpha ranges in the *Shank2*$^{-/-}$ mPFC.
(a–c) Quantification of the temporal changes in oscillatory activities in the *Shank2*$^{-/-}$ mPFC, using spectral power densities before and after nose poke (-1.5 to -0.5 sec and 0.5 to 1.5 sec, respectively). Data: minimal, maximal, median, 25%, and 75% values. Data: mean ± SEM. (n = 52 experiments from 5 WT mice, and 53 experiments from 5 *Shank2*$^{-/-}$ mice, *p < 0.05, **p < 0.01, ***p < 0.001, ns, not significant, two-way ANOVA with Tukey's test).

See Source Data for raw data values and Supplementary Table 1 for statistical details.

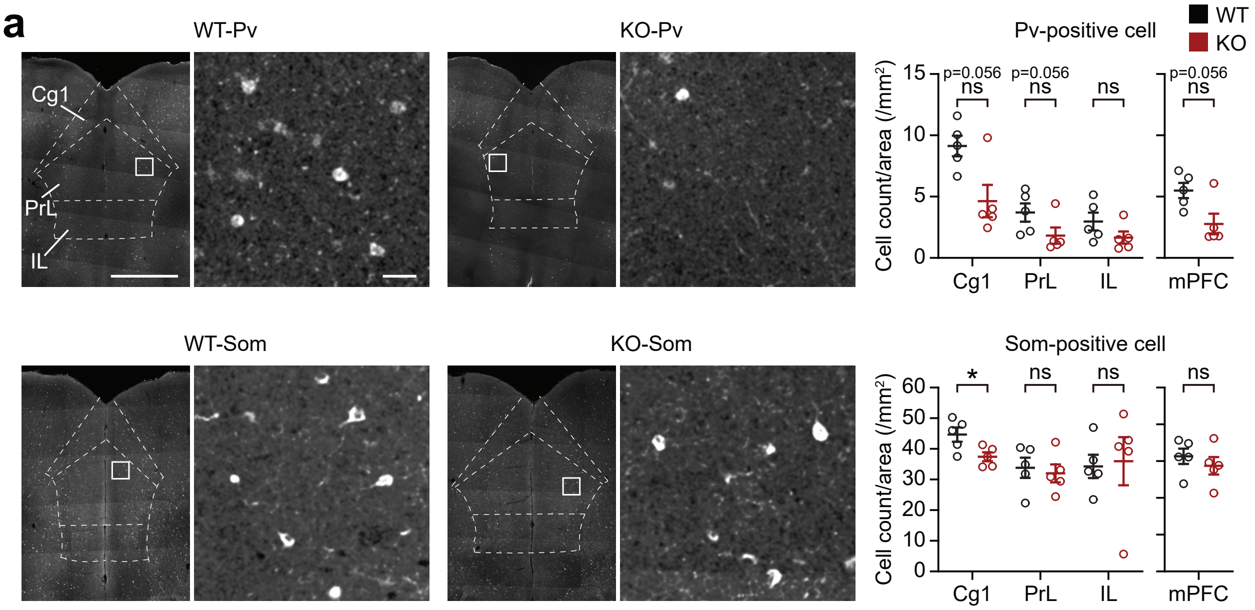

Supplementary figure 5. Normal Pv neuronal density in the *Shank2*$^{-/-}$ mPFC.
(a) Normal density of Pv- and somatostatin/Som-positive neurons in the *Shank2*$^{-/-}$ mPFC, although there is a tendency for a decrease for Pv neruons, as shown by the density of Pv/Som-positive neurons normalized to the indicated subregions of the mPFC (anterior cingular [Cg1], prelimbic [PrL], and infralimbic [IL]). The relative densities of Pv and Som neurons could vary depending the affinity of the antibodies used, although a previous study has reported that Pv and Som neurons comprise ~40% and 30% of neocortical interneurons [3]. Scale bar, 1 mm and 50 μm. Data: minimal, maximal, median, 25%, and 75% values. Data: mean ± SEM. (n = 33 slices from 5 mice [WT-PV], 36/5 [KO-PV], 33/5 [WT-SST], 36/5 [KO-SST], ns, not significant, Mann-Whitney test and Student's t-test depending the normality of data distribution).

See Source Data for raw data values and Supplementary Table 1 for statistical details.

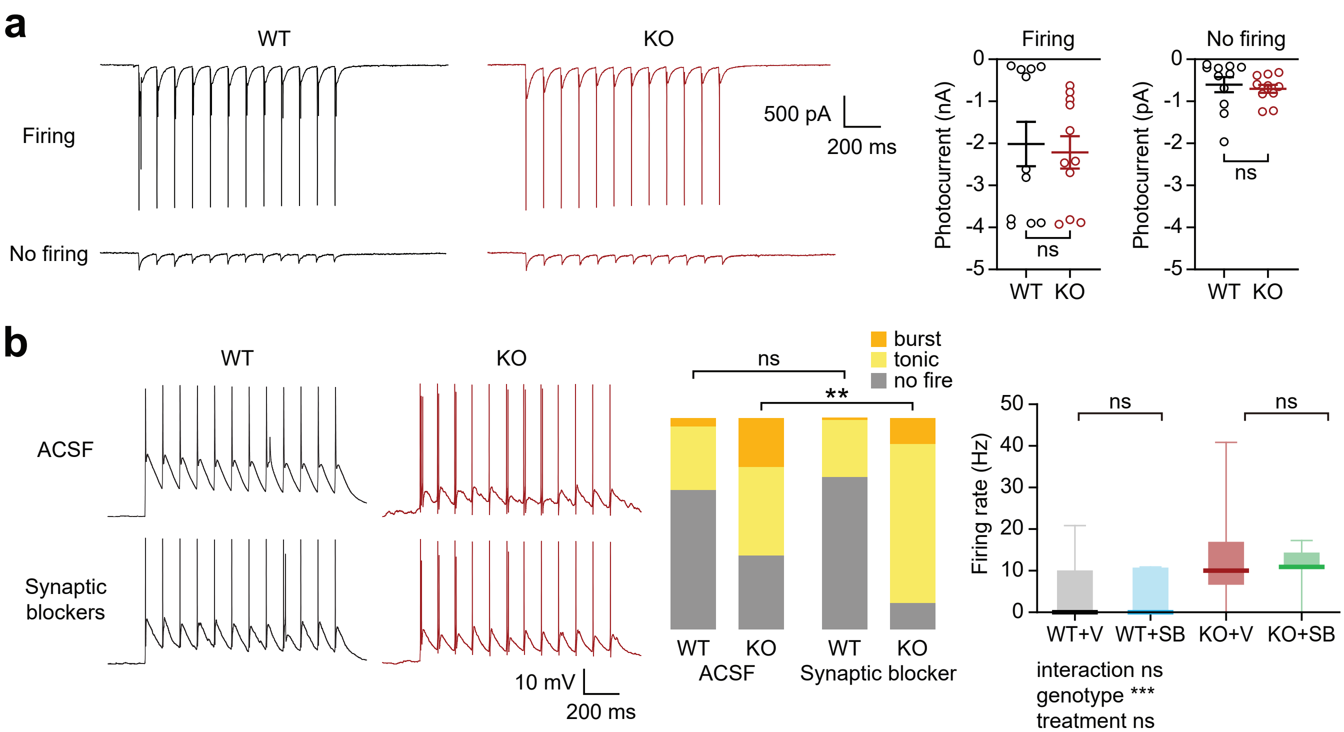

Supplementary figure 6. Comparable photocurrents in WT and *Shank2*$^{-/-}$ Pv neurons, and minimal effects of synaptic blockade on the WT-KO difference in burst firing.

(a) Comparable photocurrents recorded in Pv neurons directly light-stimulated in voltage-clamp recordings (-70 mV) in WT and *Shank2*$^{-/-}$ with in the presence/absence of induced firings. (n = 11 neurons from 3 mice [WT and KO with firing], 11 neurons from 3 mice [WT and KO without firing], ns, not significant, Mann Whitney test [with firing], Student's t-test [without firing]).

(b) Synaptic (excitatory and inhibitory) blockade minimally affects the difference in burst firing between WT and *Shank2*$^{-/-}$ Pv neurons neighboring the directly stimulated Pv neurons, as shown by neuronal proportions with distinct firing patterns and mean firing rates. The following synaptic blockers were used to block excitatory and inhibitory synaptic transmission; GABAzine (SR-95531; 10 μM) for GABA receptors, APV (50 μM) for NMDA receptors, and NBQX (10 μM) for AMPA receptors. Note that the moderate (~2-fold) decrease in burst firing in *Shank2*$^{-/-}$ Pv neurons contrasts sharply with the mefloquine-induced strong (~20-fold; from 16% to 0.67%) decrease in burst firing in *Shank2*$^{-/-}$ Pv neurons (**Fig. 5b**). Data: minimal, maximal, median, 25%, and 75% values. (n = 47/10 [WT-ACSF], 42/15 [KO-ACSF], 8/3 [WT-blockade], and 8/3 [KO-blockade], **p < 0.01, ns, not significant, Chi-square test [proportion], two-way ANOVA with Tukey's test [firing rate]).

See Source Data for raw data values and Supplementary Table 1 for statistical details.

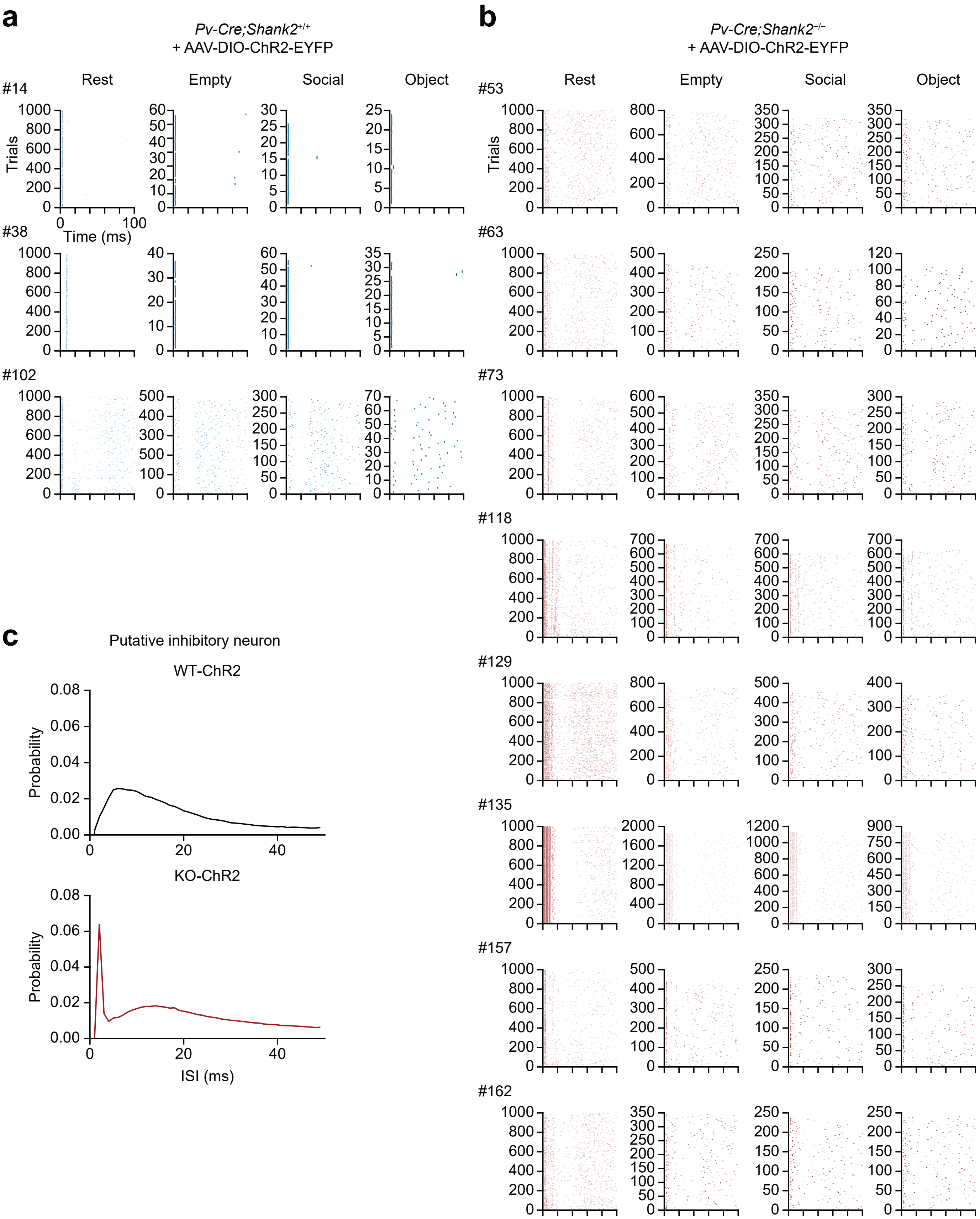
a
Pv-Cre;Shank2+/+
+ AAV-DIO-ChR2-EYFP
Rest
Empty
Social
Object
#14
#38
#102
Trials
Time (ms)
b
Pv-Cre;Shank2−/−
+ AAV-DIO-ChR2-EYFP
#53
#63
#73
#118
#129
#135
#157
#162
c
Putative inhibitory neuron
WT-ChR2
KO-ChR2
Probability
ISI (ms)

Supplementary figure 7. Optogenetic Pv neuronal stimulation induces strong burst firings in the mPFC of freely behaving *Shank2*$^{–/–}$ mice.

(a and b) Optogenetic Pv neuronal stimulation (473 nm, 5 ms, 10 Hz) leads to stronger burst firings in the mPFC of *Shank2*$^{–/–}$ mice compared with WT mice, as shown by neuronal firings that frequently occur immediately after the 5-ms light stimulation epochs. By comparison, such firings are rarely observed in the WT mPFC, where light stimulation-matched tonic firings are prevalent. *Pv-Cre;WT* and *Pv-Cre;Shank2*$^{–/–}$ mice were injected with AAV-DIO-ChR2-EYFP in the mPFC and allowed to express ChR2-EYFP for 3 week before Pv neuronal stimulation in the mPFC during R state or E/O/S encounters. Numbers indicate individual neurons.

(c) ISI (interspike interval) analyses of pInh neuronal firings in the mPFC of WT and *Shank2*$^{–/–}$ mice indicate strong increases in the ISI range smaller than 5 ms in *Shank2*$^{–/–}$ mice, which is absent in WT mice, in line with the increased burst firings in *Shank2*$^{–/–}$ Pv neurons.

See Source Data for raw data values.

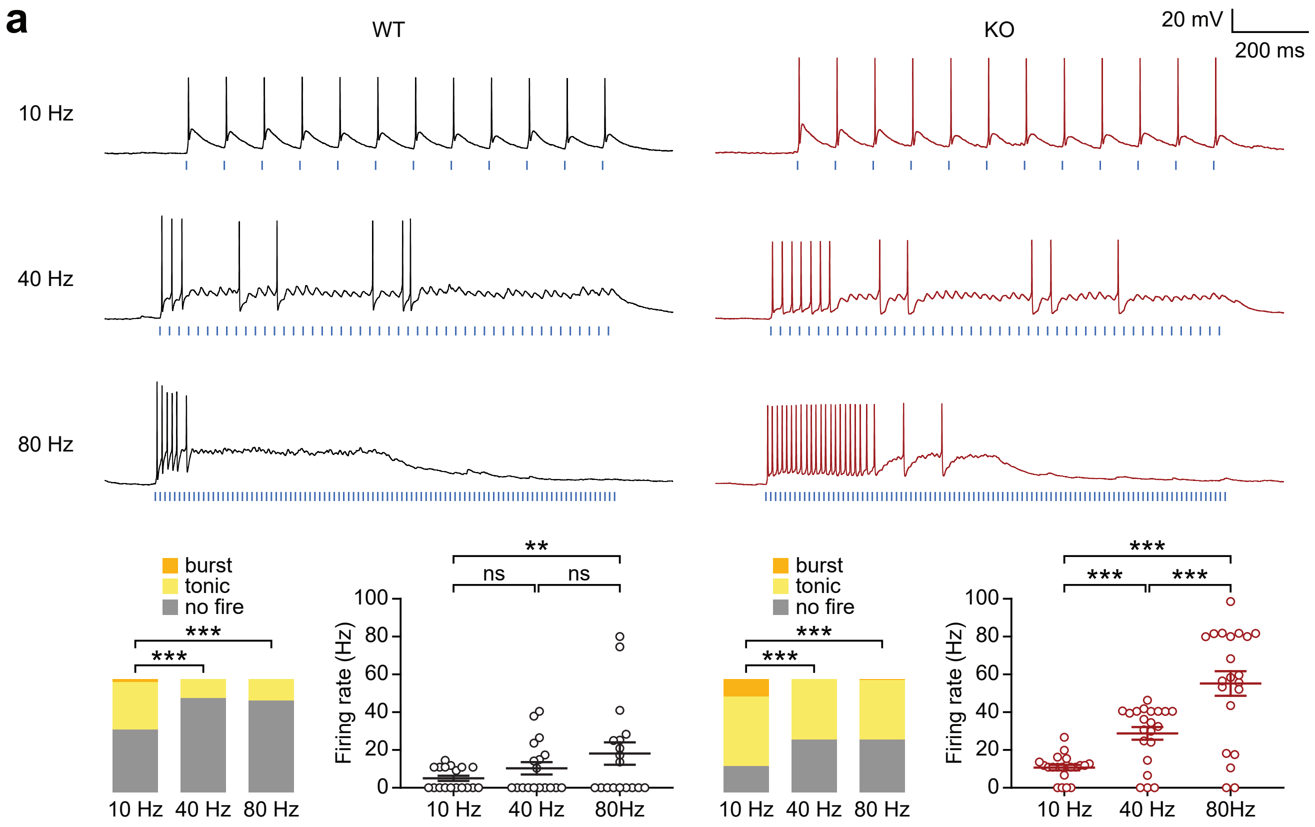

Supplementary figure 8. Differential effects of the frequency of Pv neuronal stimulation (10, 40, and 80 Hz) on burst firing.

(a) Differential effects of Pv neuronal stimulation at three sequential stimulation frequencies (10, 40, and 80 Hz) on burst firing in Pv neurons neighboring a stimulated neuron, as shown by proportions of firing patterns and mean-firing rates. Note that 10-Hz, but not 40-Hz or 80-Hz stimulation, can reliably induce burst firing in neighboring Pv neurons. Data: mean ± SEM. (n = 18 neurons from 4 mice [WT], 21/4 [KO], **p < 0.01, ***p < 0.001, ns, not significant, Chi-square test [proportion], two-way ANOVA with Tukey's test [firing rate]).

See Source Data for raw data values and Supplementary Table 1 for statistical details.

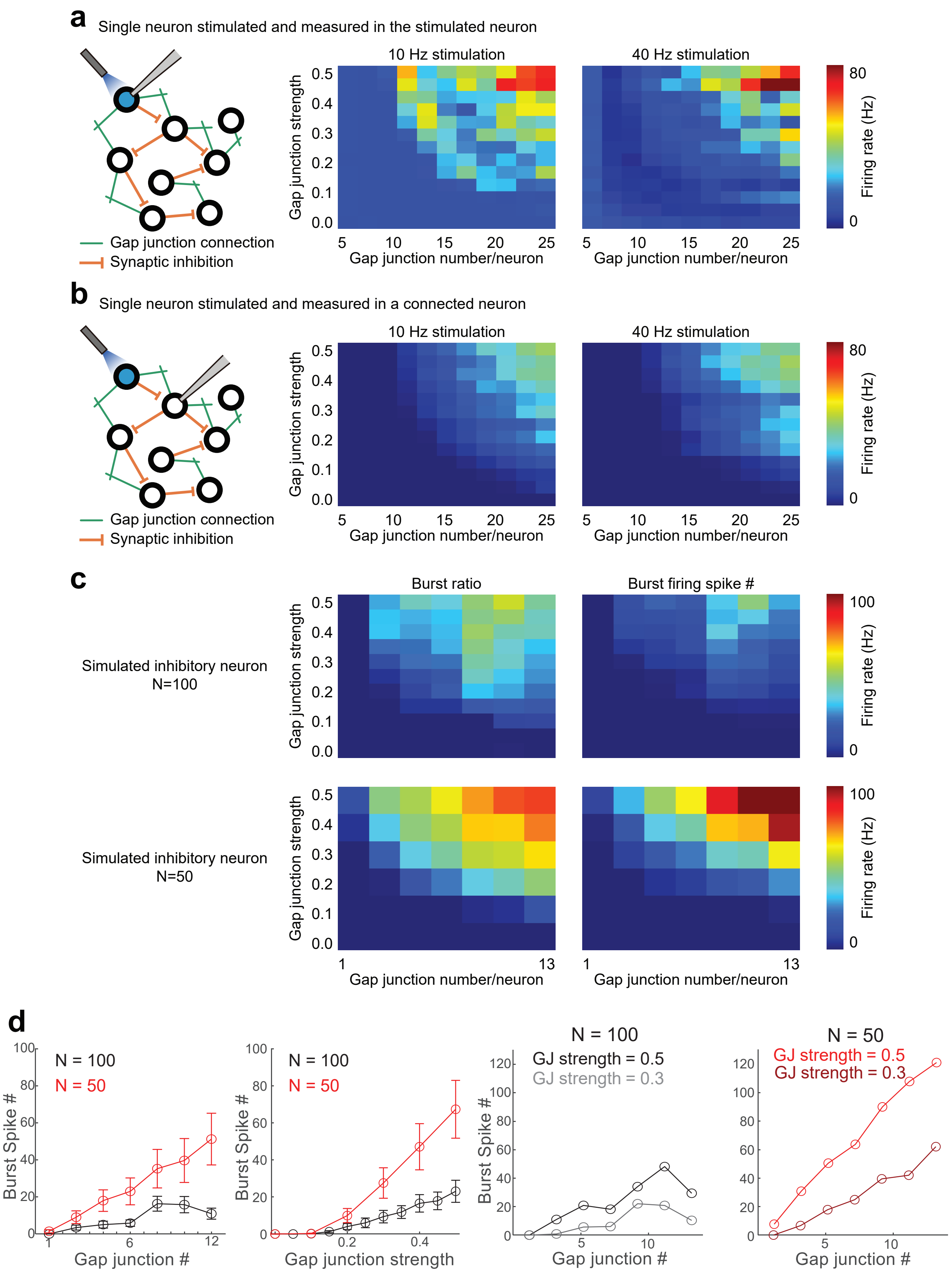
a
Single neuron stimulated and measured in the stimulated neuron
10 Hz stimulation
40 Hz stimulation
Gap junction strength
Gap junction number/neuron
Firing rate (Hz)
Gap junction connection
Synaptic inhibition
b
Single neuron stimulated and measured in a connected neuron
10 Hz stimulation
40 Hz stimulation
c
Burst ratio
Burst firing spike #
Simulated inhibitory neuron
N=100
Simulated inhibitory neuron
N=50
d
N = 100
N = 50
Burst Spike #
Gap junction #
Gap junction strength
GJ strength = 0.5
GJ strength = 0.3

Supplementary figure 9. Computational modeling of burst-firing patterns of Pv neurons induced by single Pv neuronal stimulation at 10 and 40 Hz
(a and b) Mean-firing rates in light-stimulated (at 10/40 Hz) single Pv neurons (a) and Pv neurons neighboring a light-stimulated single Pv neuron (b) in the network of 100 Pv neurons. Note that the number of gap junctions and the strength of individual gap junctions in a Pv neuron are positively correlated with the mean firing rate in the network of Pv neurons. Note also that 10- and 40-Hz Pv neuronal stimulations induce similar levels of increases in mean firings rates in stimulated and neighboring Pv neurons, although to a lesser extent in stimulated Pv neurons, in contrast with the strong burst firings induced by 10- but not 40-Hz Pv neuronal stimulation.

(c and d) Increased burst firing in the Pv neuronal network with the decreased total number of Pv neurons but the unaltered total number of gap junctions and the strength of individual gap junctions. (c) Stronger burst firing, as shown by burst ratio, in the Pv neuronal network with the decreased total number of Pv neurons (n = 50, bottom), compared with that with 100 Pv neurons (top, the condition used for the modeling described in **Fig. 5a** and the panels above [a and b]). (d) Line drawing of the results from the modeling described in (c) to highlight the impacts of the decrease in the total number of Pv neurons on burst firing (top), and the increases in the number of GJs and the strength of individual GJs on burst spike number (bottom). Data: mean ± SEM.

See Source Data for raw data values.

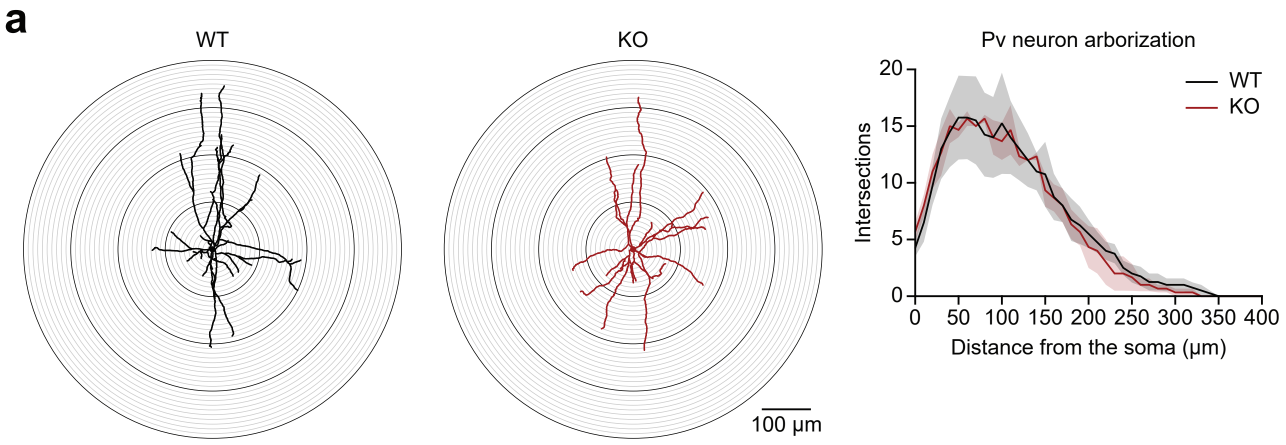

Supplementary figure 10. Normal levels of dendritic arborization in *Shank2*$^{-/-}$ Pv neurons.

(a) Comparable level of dendritic arborization in WT and *Shank2*$^{-/-}$ Pv neurons in the mPFC (9 wees) as analyzed by neuronal dye infusion (NEUROBIOTIN Tracer; Vector Lab) followed by Sholl analysis (right).

See Source Data for raw data values and Supplementary Table 1 for statistical details.

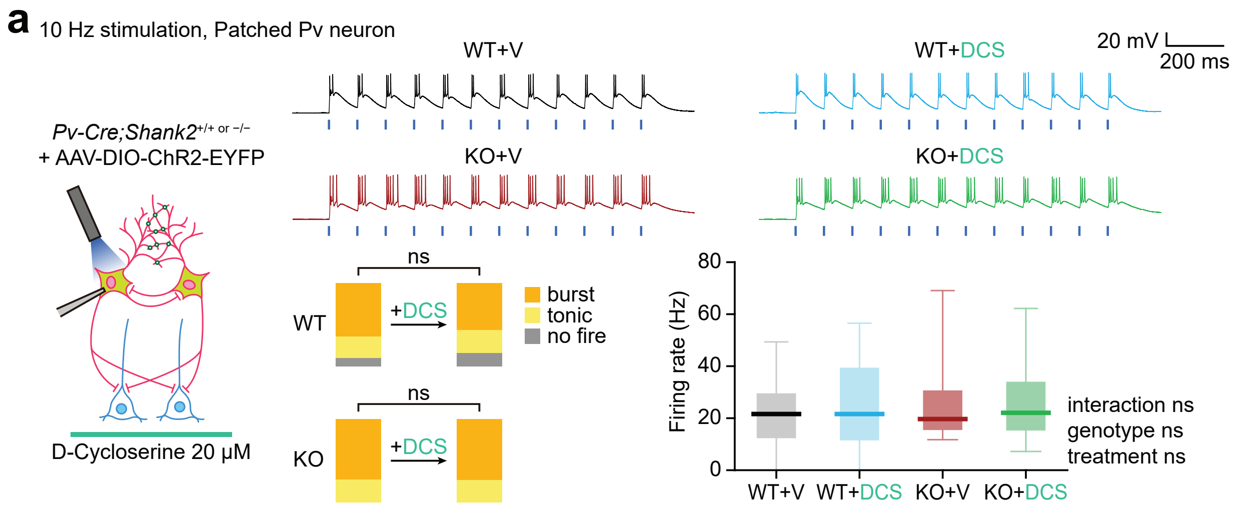

Supplementary figure 11. NMDAR activation does not enhance burst firing in directly light-stimulated WT or Shank2-KO Pv neurons.

(a) NMDAR activation by D-cycloserine treatment (20 μM) does not enhance burst firing in *Shank2*$^{-/-}$ or WT Pv neurons that are directly light stimulated in slice preparations (prelimbic, layer 2/3, 12–13 weeks). Data: minimal, maximal, median, 25%, and 75% values. (n = 20, 7 [WT-V/vehicle, before D-cycloserine/DCS treatment]; 20, 7 [WT-DCS, after DCS treatment], 16, 4 [KO-V], and 16, 4 [KO-MQ], ns, not significant, Chi-square test [proportion], Wilcoxon matched-pairs signed rank test and Student's t-test [firing rate]).

See Source Data for raw data values and Supplementary Table 1 for statistical details.

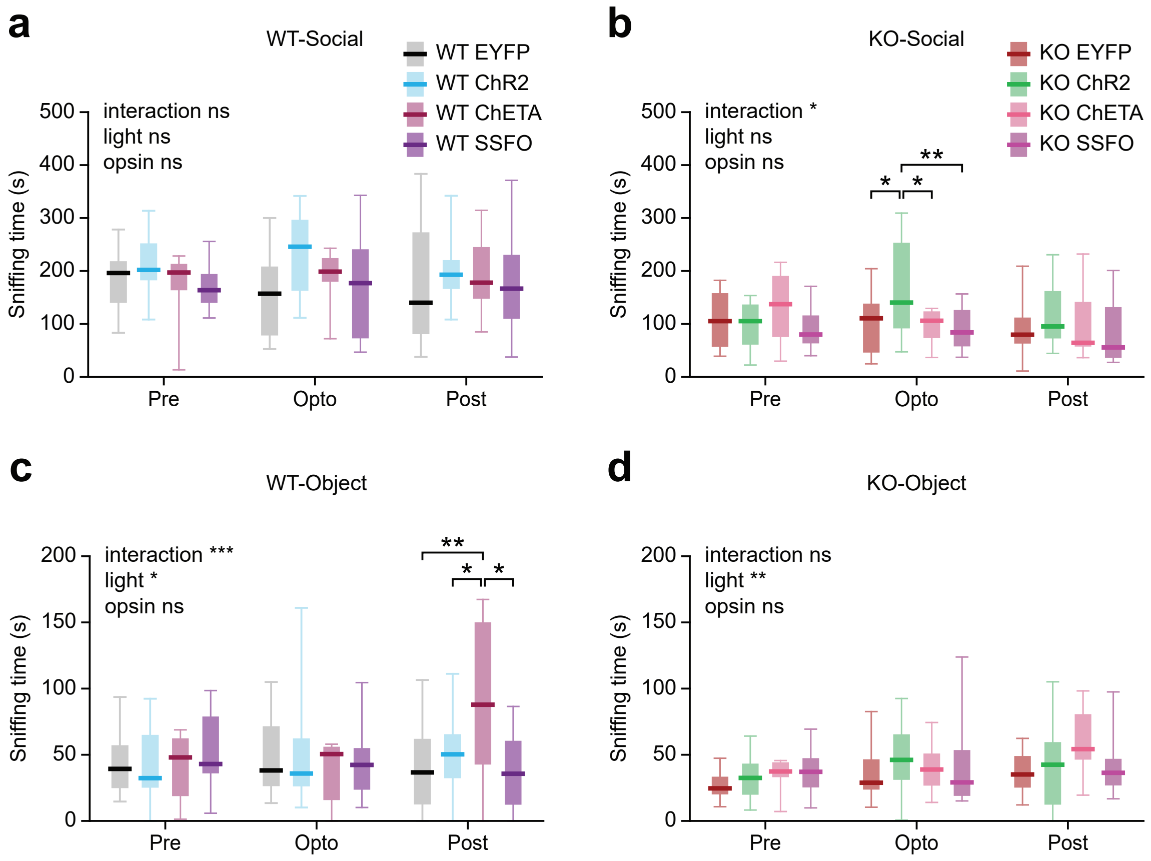

Supplementary figure 12. Burst-evoking Pv neuronal stimulation improves social interaction and cortical social representation, indicated by two-way ANOVA results.

(a–d) The results shown in Fig. 7b-e are presented here again using two-way ANOVA results. Data: minimal, maximal, median, 25%, and 75% values. Data: mean ± SEM. (n = 15 mice [WT-EYFP], 11 [KO-EYFP], 20 [WT-ChR2-10-Hz], 16 [KO-ChR2-10-Hz], 8 [WT-ChETA], 10 [KO-ChETA], 15 [WT-SSFO], 14 [KO-SSFO], *$p < 0.05$, **$p < 0.01$, ***$p < 0.001$, two-way repeated-measures ANOVA with Tukey's test).

See Source Data for raw data values and Supplementary Table 1 for statistical details.

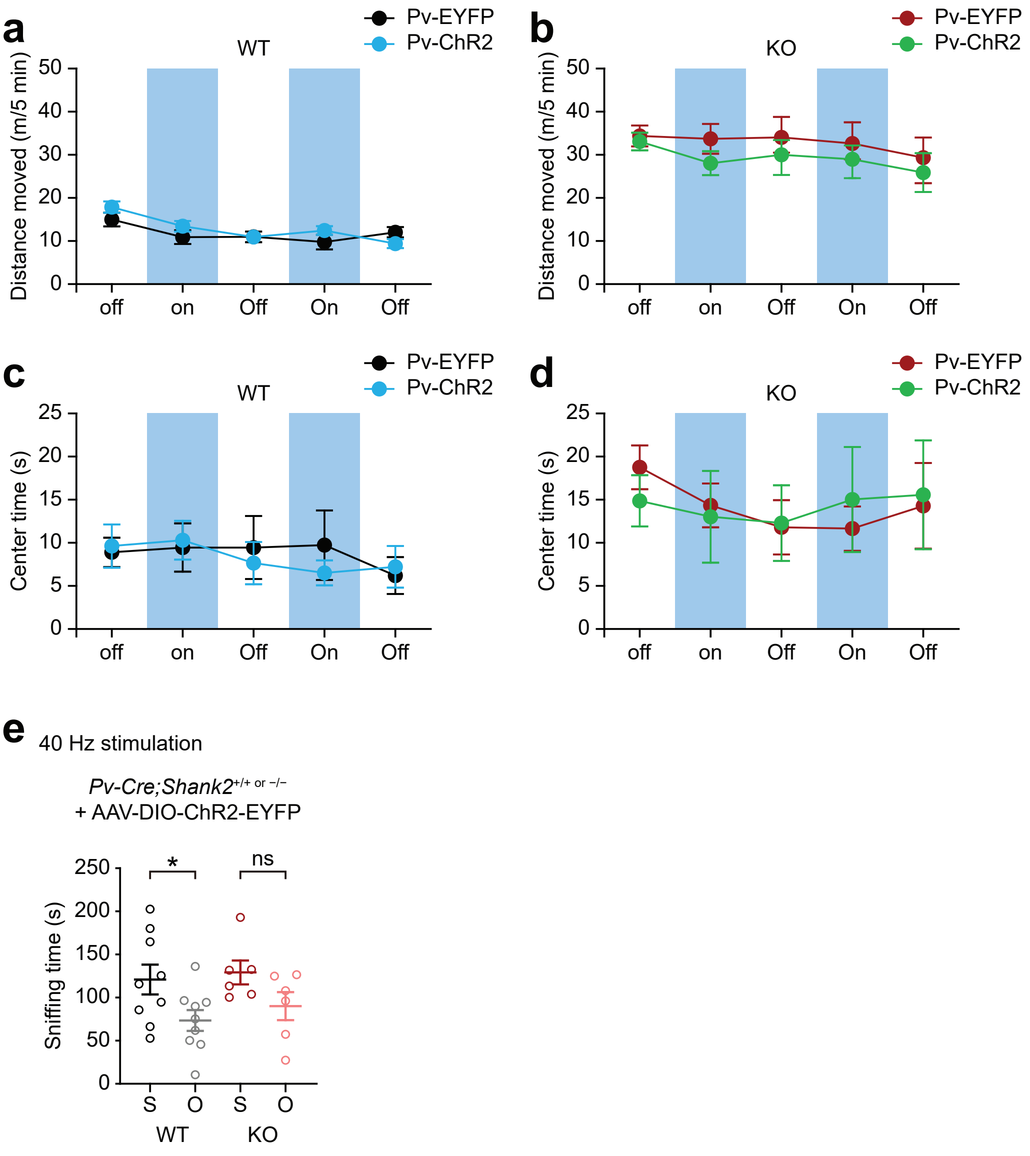
a
WT
Pv-EYFP
Pv-ChR2
Distance moved (m/5 min)
0
10
20
30
40
50
off
on
Off
On
Off
b
KO
Pv-EYFP
Pv-ChR2
Distance moved (m/5 min)
c
WT
Center time (s)
0
5
10
15
20
25
d
KO
Center time (s)
e
40 Hz stimulation
Pv-Cre;Shank2+/+ or −/−
+ AAV-DIO-ChR2-EYFP
Sniffing time (s)
0
50
100
150
200
250
*
ns
S
O
S
O
WT
KO

Supplementary figure 13. 10-Hz Pv neuronal stimulation does not affect locomotor activity or anxiety-like behavior in WT or *Shank2*$^{-/-}$ mice, and 40-Hz Pv neuronal stimulation fails to rescue social interaction in *Shank2*$^{-/-}$ mice.

(a and b) Effect of optogenetic stimulation of Pv neurons on locomotor activity. *PvCre;WT* and *Pv-Cre;Shank2*$^{-/-}$ mice were infected in the mPFC with AAV-DIO-ChR2EYFP or AAV-DIO-EYFP (>8 + 3 weeks), followed by blue light stimulation (10 Hz, 5 ms, 473 nm) in the mPFC in the open-field test, in which 5-min light-on and light-off sessions were alternated over 25 min. Data: mean ± SEM. (n = 12 mice [WT-EYFP], 20 [WT-ChR2], 12 [KO-EYFP], 14 [KO-ChR2], two-way repeated-measures ANOVA).

(c and d) Time spent in the center region of the open-field arena was also analyzed as a measure of anxiety-like behavior. Data: mean ± SEM. (n = 12 mice [WT-EYFP], 20 [WT-ChR2], 12 [KO-EYFP], 14 [KO-ChR2], two-way repeated-measures ANOVA).

(e) ChR2-driven Pv neuronal stimulation at 40 Hz (5 ms; ChR2) in the mPFC does not improve three-chamber social interaction in Pv-Cre;*Shank2*$^{-/-}$ mice infected in the mPFC with the indicated viruses (>8 + 3 weeks), as shown by time spent sniffing (or nose-poking) social and object targets. Note that light-stimulated WT mice prefer to explore S over O target, whereas light-stimulated mutant mice do not social preference. Mice were subjected to a single round of the three-chamber test, without involving Pre or Post round without light stimulation. Data: mean ± SEM. (n = 9 mice [KO-EYFP], 6 [KO-ChR2-40-Hz], *p < 0.05, ns, not significant, Student's t-test).

See Source Data for raw data values and Supplementary Table 1 for statistical details.

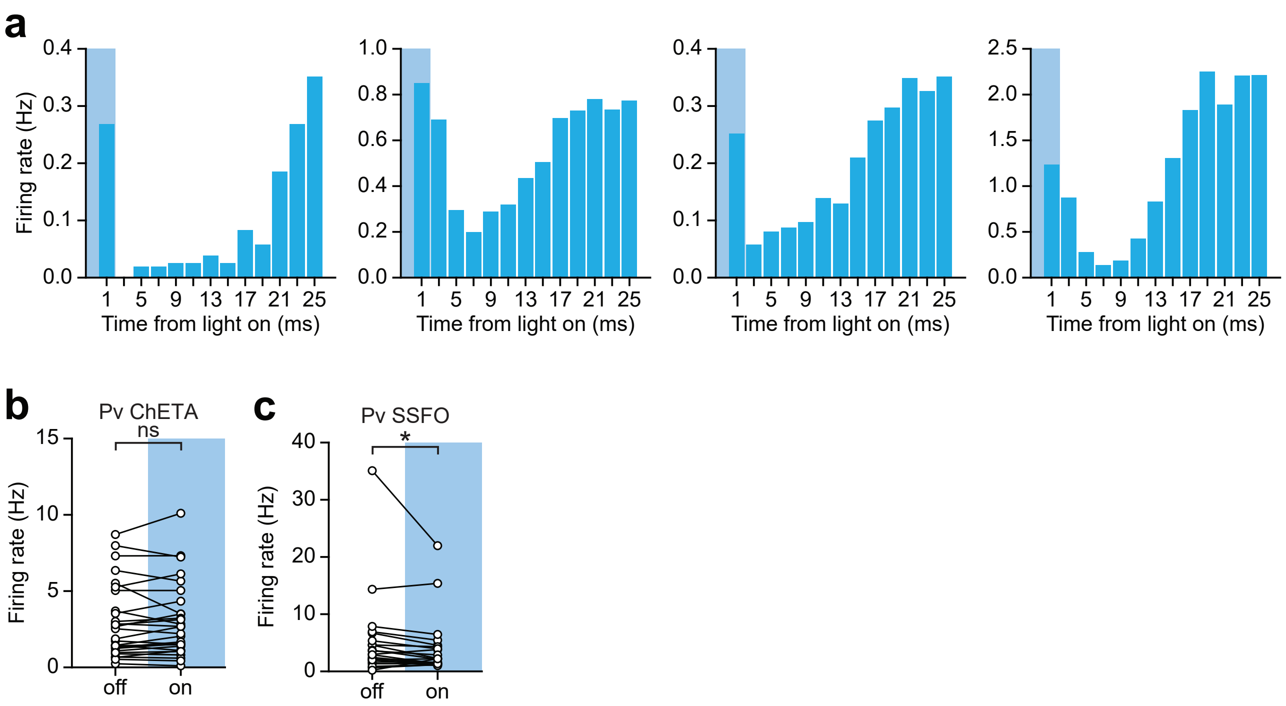

Supplementary figure 14. Examples of mPFC neuronal responses to different optogenetic manipulations of Pv neurons.
(a and b) Stimulation of ChETA-expressing Pv neurons at 40 Hz induces transient (~10–20 ms) decreases in firing rates of mPFC neurons, which likely represent Pv neuronal targets, immediately after the 2-ms light stimulation, as shown by peristimulus time histograms, without affecting the mean firing rate of mPFC neurons. In vivo single units were recorded for 20 min in mPFC neurons in *Pv-Cre;WT* mice injected in the mPFC with AAV-DIO-ChETA-EYFP in the presence and absence of light stimulation (40 Hz, 2 ms). (n = 25 neurons, ns, not significant, Wilcoxon matched-pairs signed rank test).

(c) Stimulation of Pv neurons expressing SSFO induces a decrease in the mean firing rate of mPFC neurons. In vivo single units were recorded in mPFC neurons for 20 min in *Pv-Cre;WT* mice injected in the mPFC with AAV-DIO-SSFO-EYFP in the absence of light stimulation or after a 2-s light stimulation, which induces long-lasting activation of SSFO. (n = 25 neurons, *p < 0.05, Wilcoxon matched-pairs signed rank test).

See Source Data for raw data values and Supplementary Table 1 for statistical details.

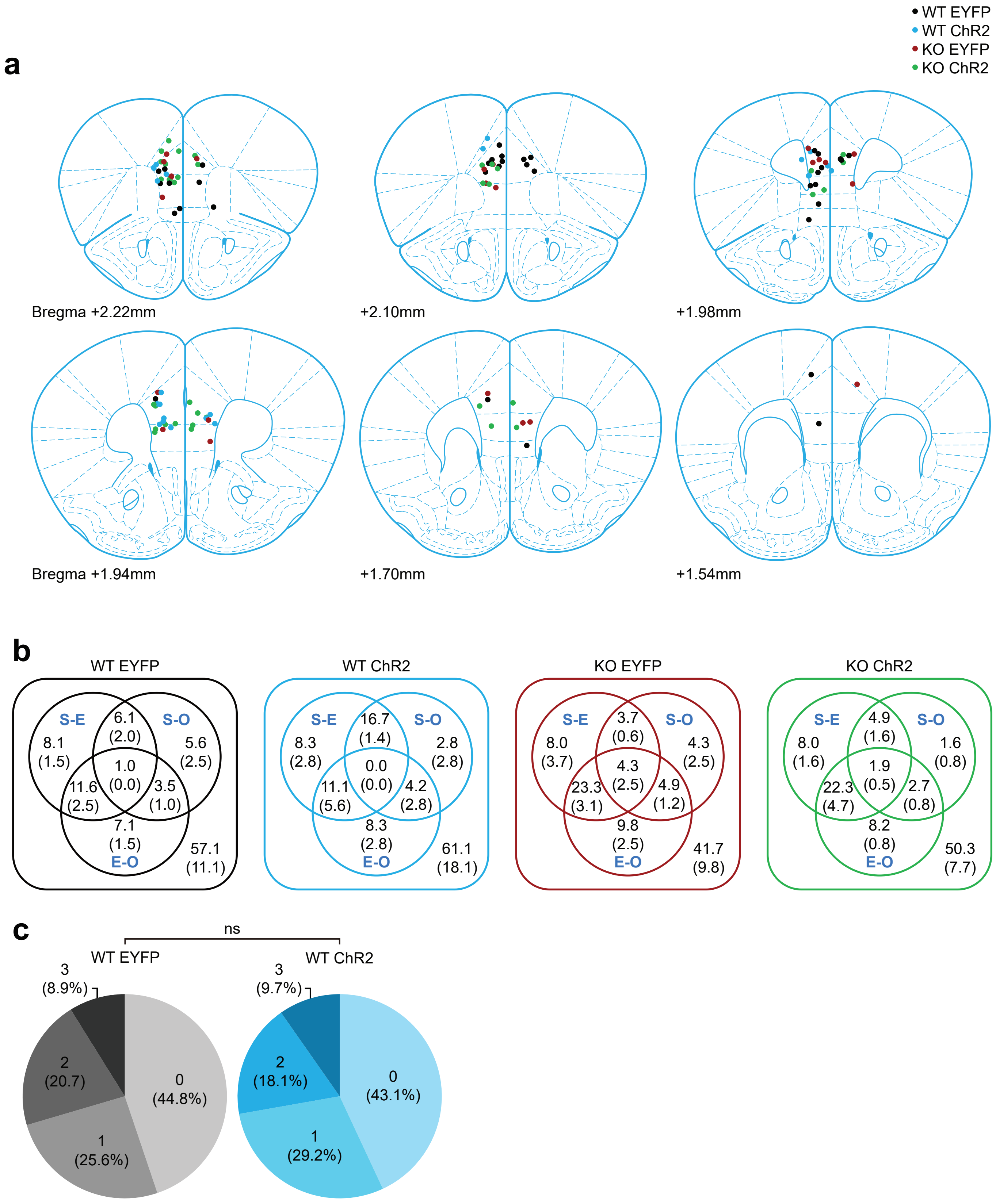

a
WT EYFP
WT ChR2
KO EYFP
KO ChR2
Bregma +2.22mm
+2.10mm
+1.98mm
Bregma +1.94mm
+1.70mm
+1.54mm
b
WT EYFP
S-E
S-O
E-O
6.1 (2.0)
8.1 (1.5)
5.6 (2.5)
1.0 (0.0)
11.6 (2.5)
3.5 (1.0)
7.1 (1.5)
57.1 (11.1)
WT ChR2
16.7 (1.4)
8.3 (2.8)
2.8 (2.8)
0.0 (0.0)
11.1 (5.6)
4.2 (2.8)
8.3 (2.8)
61.1 (18.1)
KO EYFP
3.7 (0.6)
8.0 (3.7)
4.3 (2.5)
4.3 (2.5)
23.3 (3.1)
4.9 (1.2)
9.8 (2.5)
41.7 (9.8)
KO ChR2
4.9 (1.6)
8.0 (1.6)
1.6 (0.8)
1.9 (0.5)
22.3 (4.7)
2.7 (0.8)
8.2 (0.8)
50.3 (7.7)
c
ns
WT EYFP
3 (8.9%)
2 (20.7)
0 (44.8%)
1 (25.6%)
WT ChR2
3 (9.7%)
2 (18.1%)
0 (43.1%)
1 (29.2%)

Supplementary figure 15. Positions of tetrode tips in the mPFC, and responses of WT and *Shank2*$^{–/–}$ mPFC neurons during target encounters in the presence and absence of 10-Hz optogenetic Pv neuronal stimulation.
(a) Positions of tetrode tips in the mPFC of WT and *Shank2*$^{–/–}$ mice used to measure single-unit recordings in the presence and absence of 10-Hz Pv neuronal stimulation.

(b) Discharge patterns of mPFC neurons in WT and *Shank2*$^{–/–}$ mice (3 mo) to social and non-social targets, as shown by a Venn diagram with proportions of mPFC neurons (in percentage) that show discriminative discharges (ANOVA, $p < 0.05$) to specific target pairs (S-E, S-O, and E-O) or those with target-specific responses (S-specific/spc, overlap of S-E and S-O; O-spc, overlap of S-O and E-O; E-spc, overlap of S-E and E-O). (n = 194 neurons from 7 mice for WT-EYFP, 72, 8 [WT-ChR2], 163, 4 [KO-EYFP], and 364, 9 [KO-ChR2]).

(c) 10-Hz Pv neuronal stimulation in the mPFC of WT mice exploring social and nonsocial targets in the linear apparatus does not affect the number of total targets to which a single neuron can respond. (n = 194, 7 [WT-EYFP], 72, 8 [WT-ChR2], ns, not significant, Chi-square test).

See Source Data for raw data values and Supplementary Table 1 for statistical details.

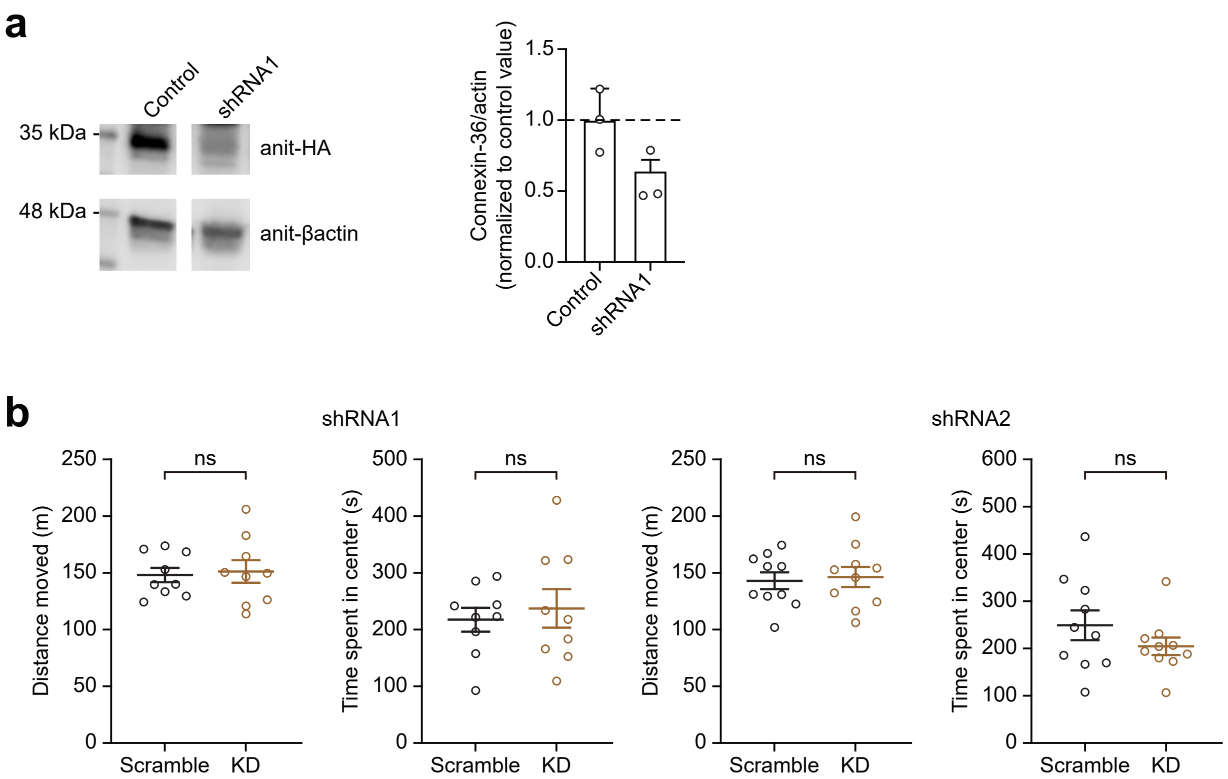

Supplementary figure 16. Characterization of connexin-36 knockdown constructs. (a) Knockdown efficiency of connextin-36-shRNA1 and connextin-36-shRNA2 by AAV-S5E2-Flex-shRNA1 infection of HEK293T cells expressing the connexin-36-HA expression construct, followed by immunoblot analysis, which show that ~50% knockdown efficiency of target protein expression. Test of shRNA2 could not be performed because of low infection efficiency of HEK293T cells and also detecting reliable bands of endogenous connexin-36 protein in mouse brain lysates by immunoblot analysis was not feasible. (n = 3 independent experiments [WT and KO]). (b) Lack of the effects of connexin-36 knockdown (KD) by AAV-shRNA1/2 in the locomotor activity (total distance moved) and anxiety-like behavior (center time) in WT mice in the open-field test. (n = 9 mice [control 1], 9 [shRNA1], 10 [control 2], 10 [shRNA2], ns, not significant, Student's t-test).

See Source Data for raw data values and images and Supplementary Table 1 for statistical details.

Supplementary tables

Supplementary Table 1. Statistical details.

Figure	Assay Performed	Paremeter	Comparison			Descriptive Statistics		Statistical Analysis		
			Sex	Age	n	Average ± SEM	Median (1Q, 3Q)	Statistical Test	Significance	Summary
Figure1d	in vivo recording	Target	M	>12wks	WT=299 neurons/5 mice<br>KO=410 neurons/5 mice	WT nT n=207,T 86<br>KO nT n=255, T 174		Chi-square test<br>two-sided	p=0.0021	**
		Side			WT=295 neurons/5 mice<br>KO=409 neurons/5 mice	WT nSi n=258,Si 41<br>KO nSi n=326,Si 84		Chi-square test<br>two-sided	p=0.0194	*
		S-sepecific			WT=295 neurons/5 mice<br>KO=409 neurons/5 mice	WT nS-spc n=285, S-spc 14<br>KO nS-spc n=387, S-spc 23		Chi-square test<br>two-sided	p=0.549	ns
		O-sepecific			WT=295 neurons/5 mice<br>KO=409 neurons/5 mice	WT nO-spc n=295,O-spc 4<br>KO nO-spc n=389, O-spc 21		Chi-square test<br>two-sided	p=0.007	**
		E-sepecific			WT=295 neurons/5 mice<br>KO=409 neurons/5 mice	WT nES-spc n=278, E-spc 21<br>KO nE-spc n=319, E-spc 91		Chi-square test<br>two-sided	p<0.0001	***
figure 1e	in vivo recording	number of target discrimination	M	>12wks	WT=295 neurons/5 mice<br>KO=409 neurons/5 mice	WT n=191,55,32,8,2<br>KO n=196,84,91,34,5		Chi-square tes<br>two-sided	p<0.0001	***
figure 2b	in vivo recording	putative Excitatory neuron	M	>12wks	WT n=286 /5	E-R -0.01675±0.0067<br>O-R -009406±0.007519<br>S-R -0.007818±0.008909		Friedman test with Dunn's<br>multiple comparison test	p=0.0005;<br>R vs E p=0.0005,<br>R vs O p=0.0290,<br>R vs S p=0.0072	
					KO n=340 /5	E-R -0.01957±0.006340<br>O-R -0.001944±0.007018<br>S-R -0.01962±0.006958		Friedman test with Dunn's<br>multiple comparison test	p<0.0001;<br>R vs E p=0.0109,<br>R vs O p<0.0001,<br>R vs S p=0.0030	
		putative inhibitory neuron			WT n=36 /5	E-R 0.01394±0.007035<br>O-R 0.01120±0.009400<br>S-R -0.01242±0.006205		Friedman test<br>two-sided	p=0.258	
					KO n=41 /5	E-R -0.007318±0.009572<br>O-R -0.006337±0.006772<br>S-R -0.01242±0.006205		Friedman test<br>two-sided	p=0.118	
Figure 2b	in vivo recording(pInh)	High firing ISI diff.	M	>12wks	WT n=36 /5	E-R=0.01394±0.0070;<br>O-R=0.0112±0.0094;<br>S-R=0.030±0.0099		Repeated measures of Two Way ANOVA with Sidak's mutiple comparison	genotype p=0.0129<br>target p=0.2808<br>interaction p=0.0082	WT vs KO: E-R p=0.1945, O-R p=0.350, S-R p=0.0011<br>WT:E-R vs O-R p=0.95, E-R vs S-R p=0.032, O-R vs S-R p=0.0085<br>KO:E-R vs O-R p=0.997, E-R vs S-R p=0.762, O-R vs S-R p=0.650
					KO n=41 /5	E-R=-0.0073±0.0061;<br>O-R=-0.0063±0.0068;<br>S-R=-0.0124±0.0062				
	in vivo recording(pExc)	High firing ISI diff.	M	>12wks	WT n=286 /5	E-R=-0.017±0.0067;<br>O-R=-0.0094±0.0075;<br>S-R=-0.0078±0.0089		Repeated measures of<br>Two Way ANOVA	genotype p= 0.249<br>target p=0.515<br>interaction p= 0.450	ns
					KO n=340 /5	E-R=-0.020±0.0063;<br>O-R=-0.019±0.0070;<br>S-R=-0.020±0.0070				
Figure 3a	Linear chamber	Delta PSD	M	12wks	WT n=53<br>KO n=52	Rest:0.335±0.00912<br>Target:0.321±0.00527<br>Rest:0.366±0.00921<br>Target:0.3581±0.00532		Two Way ANOVA with Tukey's test<br>F(1,416)=21.279	genotype p<0.001<br>event p=0.153<br>interaction p=0.712	rest WT vs KO p=0.014<br>target WT vs KO p<0.001<br>WT rest vs target p>0.05<br>KO rest vs target p>0.05
		Theta PSD			WT n=53<br>KO n=52	Rest: 0.0453±0.00318<br>Target:0.0362±0.00184<br>Rest: 0.0768±0.00321<br>Target: 0.0465±0.00185		Two Way ANOVA with Tukey's test<br>F(1,416)=64.153	genotype p<0.001<br>event p<0.001<br>interaction p<0.001	rest WT vs KO p<0.001<br>target WT vs KO p<0.001<br>WT rest vs target p=0.014<br>KO rest vs target p<0.001
		Alpha PSD			WT n=53<br>KO n=52	Rest: 0.0158±0.00177<br>Target: 0.0138±0.00102<br>Rest:0.028±0.00179<br>Target: 0.0168±0.00103		Two Way ANOVA with Tukey's test<br>F(1,416)=64.153	genotype p<0.001<br>event p<0.001<br>interaction p=0.002	rest WT vs KO p<0.001<br>target WT vs KO p=0.039<br>WT rest vs target p=0.331<br>KO rest vs target p<0.001
		Gamma PSD			WT n=53<br>KO n=52	Rest:0.0164±0.00284<br>Target:0.0155±0.00164<br>Rest:0.0221±0.00287<br>Target:0.0125±0.00166		Two Way ANOVA with Tukey's test<br>F(1,416)=5.047	genotype p=0.548<br>event p=0.025<br>interaction p=0.062	rest WT vs KO p=0.153<br>target WT vs KO p=0.204<br>WT rest vs target p=0.79<br>KO rest vs target p=0.004
Figure 3c	Linear Chamber	WT pre,post-Gamma PSD	M	12wks	Empty WT=52<br>Social WT=52<br>object WT=52	Pre:0.0143±0.000620<br>Post:0.0139±0.000620<br>Pre:0.0143±0.000620<br>Post:0.0139±0.000620<br>Pre:0.0143±0.000620<br>Post:0.0139±0.000620		Two Way ANOVA with Tukey's test<br>F(1,104)=4.907	time p=0.441<br>target p=0.009<br>interaction p=0.031	pre E vs S p=0.045<br>post E vs S p=0.003<br>Empty pre vs post p=0.677<br>Social pre vs post p=0.258<br>object pre vs post p=0.023
		KO pre,post-Gamma PSD	M	12wks	Empty KO=53<br>Social KO=53<br>object KO=53	Pre:0.0143±0.000620<br>Post:0.0139±0.000620<br>Pre:0.0143±0.000620<br>Post:0.0139±0.000620<br>Pre:0.0143±0.000620<br>Post:0.0139±0.000620		Two Way ANOVA with Tukey's test<br>F(1,102)=14.22	time p<0.001<br>target p=0.149<br>interaction p=0.346	pre E vs S p>0.05<br>post E vs S p>0.05<br>Empty pre vs post p=0.003<br>Social pre vs post p=0.005<br>object pre vs post p<0.001
Figure 4a	slice electrophysiology	sEPSC Py frequency	M	12wks	WT : 23 neurons / 5 mice<br>KO : 14 neurons / 4 mice	WT: 3.098±0.2391<br>KO: 2.893±0.2716	2.95<br>(2.200, 3.483)<br>2.738<br>(2.265, 3.856)	Mann Whitney, two-tailed	p=0.8167	ns
	slice electrophysiology	sEPSC Py amplitude	M	12wks	WT : 23 neurons / 5 mice<br>KO : 14 neurons / 4 mice	WT: 15.4±0.334<br>KO: 16.41±4.516	15.29<br>(13.89, 16.79)<br>13.16<br>(14.24, 16.88)	Mann Whitney, two-tailed	p=0.76708	ns
Figure 4b	slice electrophysiology	sIPSC Py frequency	M	12wks	WT : 19 neurons / 6mice<br>KO : 18 neurons / 4mice	WT: 2.726±0.4253<br>KO: 3.088±0.5006	2.2<br>(1.290, 4.070)<br>2.6<br>(1.988, 3.583)	Mann Whitney, two-tailed	p=0.4079	ns
	slice electrophysiology	sIPSC Py amplitude	M	12wks	WT : 19 neurons / 6mice<br>KO : 18 neurons / 4mice	WT: 52.79±4.473<br>KO: 48.42±3.274	45.44<br>(39.13, 59.61)<br>48.8<br>(36.22, 57.14)	Mann Whitney, two-tailed	p=0.7986	ns
Figure 4c	slice electrophysiology	Intrinsic excitability IO curve Py	M	>12wks	WT : 21 neurons / 5mice<br>KO : 13 neurons / 5mice	WT:3.750±3.254<br>KO:2.630±1.163<br>WT:4.536±1.334<br>KO:7.746±1.674<br>WT:10.927±1.874<br>KO:11.081±2.359<br>WT:15.931±2.138<br>KO:15.209±2.716<br>WT:20.839±2.099<br>KO:18.523±3.101<br>WT:25.600±2.276<br>KO:21.341±3.278<br>WT:29.850±2.548<br>KO:23.794±3.630<br>WT:33.742±2.747<br>KO:27.428±3.365<br>WT:37.146±3.108<br>KO:30.000±3.574<br>WT:42.270±3.271<br>KO:31.847±3.846	WT:0.000(0.000,68.027)<br>KO:0.000(0.000,10.745)<br>WT:0.000(0.000,15.300)<br>KO:9.443(0.000,16.625)<br>WT:10.808(0.000,26.664)<br>KO:12.747(0.000,22.449)<br>WT:14.791(0.000,33.259)<br>KO:18.427(0.000,26.727)<br>WT:19.960(7.935,38.416)<br>KO:23.623(0.000,31.578)<br>WT:25.136(11.432,42.599)<br>KO:28.783(0.000,34.157)<br>WT:29.899(9.056,46.804)<br>KO:31.021(0.000,39.344)<br>WT:34.442(11.042,52.854)<br>KO:33.116(6.920,45.948)<br>WT:39.228(12.788,61.671)<br>KO:34.148(9.124,51.546)<br>WT:43.122(15.566,66.390)<br>KO:33.460(9.094,56.216)	Repeated measures of Two Way ANOVA with Bonferroni's test	Interaction F (9, 288) = 3.705,p=0.0002<br>pA (injected current) F (1.418, 45.37) = 120.6,p<0.0001<br>Group (WT/KO) F (1, 32) = 1.026,p=0.3187	WT vs. KO<br>50: P>0.9999<br>100: P>0.9999<br>150: P>0.9999<br>200: P>0.9999<br>250: P>0.9999<br>300: P>0.9999<br>350: P>0.9999<br>400: P>0.9999<br>450: P>0.9999<br>500: P=0.4867
Figure 4d	slice electrophysiology	sIPSC Pv frequency	M	12wks	WT : 23 neurons / 7mice<br>KO : 14 neurons / 3mice	WT: 3.09±0.2391<br>KO: 2.893±0.2761	2.95<br>(1.430, 3.840)<br>2.893<br>(2.223, 4.713)	Mann Whitney, two-tailed	p=0.6708	ns
	slice electrophysiology	sIPSC Pv amplitude	M	12wks	WT : 23 neurons / 7mice<br>KO : 14 neurons / 3mice	WT: 15.40±0.3334<br>KO: 16.41±1.207	15.19<br>(29.64, 44.83)<br>16.41<br>(30.01, 41.06)	Mann Whitney, two-tailed	p=0.8167	ns
Figure 4e	slice electrophysiology	sIPSC Pv frequency	M	12wks	WT : 23 neurons / 6mice<br>KO : 28 neurons / 8mice	WT: 2.847±0.3463<br>KO: 3.820±0.4004	2.25<br>(1.430, 3.840)<br>3.485<br>(2.223, 4.713)	Mann Whitney, two-tailed	p=0.0912	ns
	slice electrophysiology	sIPSC Pv amplitude	M	12wks	WT : 23 neurons / 6mice<br>KO : 28 neurons / 8mice	WT: 37.77±1.957<br>KO: 36.00±1.547	37.99<br>(29.64, 44.83)<br>34.65<br>(30.01, 41.06)	Unpaired t test, two-tailed	p=0.4752	ns
Figure 4f	slice electrophysiology	Intrinsic excitability IO curve Pv	M	>12wks	WT : 24 neurons / 6mice<br>KO : 19 neurons / 4mice	WT:0.000±0.000<br>KO:0.509±0.509<br>WT:0.000±0.000<br>KO:2.473±1.386<br>WT:7.039±5.094<br>KO:4.784±2.286<br>WT:17.299±5.299<br>KO:21.556±6.656<br>WT:43.303±4.907<br>KO:30.505±6.611<br>WT:56.997±5.119<br>KO:54.505±8.782<br>WT:73.447±5.570<br>KO:72.001±7.591<br>WT:84.004±6.251<br>KO:81.947±6.969<br>WT:93.224±6.923<br>KO:91.488±7.687<br>WT:109.071±4.739<br>KO:99.811±8.485	WT:0.000(0.000,0.000)<br>KO:0.000(0.000,9.662)<br>WT:0.000(0.000,0.000)<br>KO:0.000(0.000,19.746)<br>WT:0.000(0.000,118.694)<br>KO:0.000(0.000,33.164)<br>WT:0.000(0.000,107.914)<br>KO:8.709(0.000,107.239)<br>WT:49.456(0.000,80.352)<br>KO:25.795(0.000,96.618)<br>WT:62.506(0.000,89.397)<br>KO:53.383(0.000,156.658)<br>WT:76.824(0.000,107.316)<br>KO:67.854(21.478,153.846)<br>WT:89.165(0.000,119.662)<br>KO:79.899(22.683,150.422)<br>WT:97.175(0.000,135.886)<br>KO:97.430(24.681,160.000)<br>WT:108.004(48.780,148.209)<br>KO:108.187(26.537,165.695)	Repeated measures of Two Way ANOVA with Bonferroni's test	Interaction F (9, 369) = 0.6606,P=0.7445<br>pA (injected current) F (2.534, 103.9) = 161.2,P<0.0001<br>Group (WT/KO) F (1, 41) = 0.2196,P=0.6418	WT vs. KO<br>50: P>0.9999<br>100: P=0.9130<br>150: P>0.9999<br>200: P>0.9999<br>250: P>0.9999<br>300: P>0.9999<br>350: P>0.9999<br>400: P>0.9999<br>450: P>0.9999<br>500: P>0.9999

**Figure 4h-i**	slice electrophysiology with optogenetic stimulation	WT	F	>12wks	WT-on<br>n=9 /3<br><br>WT-off<br>n=8 /3	off:2.642±1.866	off:0.8448(0.3869, 1.292)	Repeated measures of Two Way ANOVA with Tukey's test	Interaction F(2,7)=1.929, p=0.072<br>light F(2,15)=3.485, p=0.082<br>Hz F(2,7)=367, p=<0.001	WT<br>on vs off<br>25: P > 0.05<br>75: P > 0.05<br>125: P > 0.05<br>175: P > 0.05<br>225: P < 0.05<br>275: P < 0.05<br>325: P > 0.05<br>375: P > 0.05
						on:0.3933±0.1169	on:0.4072(0.09756, 1.503)			
						off:4.959±3.256	off:1.565(1.1031, 2.859)			
						on:0.7999±0.2639	on:0.6133(0.1282, 1.503)			
						off:8.882±3.585	off:6.306(3.112, 8,543)			
						on:3.425±0.4985	on:3.360(2.221, 4.778)			
						off:16.39±3.705	off:14.52(8.653, 19.22)			
						on:9.081±1.347	on:8.720(5.269, 12.93)			
						off:25.96±3.520	off:26.22(17.74, 30.00)			
						on:18.04±1.747	on:17.42(13.53, 22.50)			
						off:32.43±3.297	off:34.27(24.56, 36.37)			
						on:24.26±1.859	on:23.79(13.53, 22.50)			
						off:36.50±2.829	off:37.08(29.50, 41.73)			
						on:29.56±1.718	on:29.09(25.98, 31.99)			
						off:41.46±2.699	off:41.31(35.24,47.52)			
						on:34.56±1.729	on:34.18(30.05, 36.06)			
		KO	F	>12wks	KO-on<br>n=17/6<br><br>KO-off<br>n=17/6	off:2.642±1.866	off:0.7860(0.4630, 1.217)	Repeated measures of Two Way ANOVA with Tukey's test	Interaction F(2,7)=0.467, p=0.858<br>light F(2,32)=0.636, p=0.431<br>Hz F(2,7)=886, p=<0.001	KO<br>on vs off<br>25: P > 0.05<br>75: P > 0.05<br>125: P > 0.05<br>175: P > 0.05<br>225: P > 0.05<br>275: P > 0.05<br>325: P > 0.05<br>375: P > 0.05
						on:0.3933±0.1169	on:0.7927(0.2297, 1.058)			
						off:4.959±3.256	off:1.918(1.371, 2.439)			
						on:0.7999±0.2639	on:1.565(0.5869, 2.063)			
						off:8.882±3.585	off:5.471(4.537, 6.821)			
						on:3.425±0.4985	on:4.935(2.712, 7.192)			
						off:16.39±3.705	off:15.33(12.65, 17.24)			
						on:9.081±1.347	on:13.88(7.746, 17.27)			
						off:25.96±3.520	off:23.84(221.23, 26.32)			
						on:18.04±1.747	on:24.14(17.10, 26.41)			
						off:32.43±3.297	off:30.50(26.04, 32.45)			
						on:24.26±1.859	on:28.69(24.16, 33.28)			
						off:36.50±2.829	off:32.97(29.32, 36.14)			
						on:29.56±1.718	on:31.20(28.49, 35.01)			
						off:41.46±2.699	off:37.54(34.40, 40.71)			
						on:34.56±1.729	on:37.01(34.52, 39.75)			
**Figure 4j-k**	slice electrophysiology	siEPSC	F	>12wks	WT off: 8 neurons / 3mice<br>WT on: 9 neurons / 3mice	WT off:0.1043±0.005252	WT off:0.102(0.06888, 0.1327)	Two-way repeated measures of ANOVA with Tukey's test	Interaction F(2,7)=1.929, p=0.072<br>light F(2,15)=3.485, p=0.082<br>Hz F(2,7)=367, p=<0.001	*
						WT on:0.07946±0.003588	WT on:0.07143(0.05102, 0.102)			
						WT off:0.1156±0.006205	WT off:0.102(0.08163, 0.1327)			*
						WT on:0.04605±0.003081	WT on:0.04082(0.02041, 0.07143)			
						WT off:0.09717±0.00528	WT off:0.09184(0.07143, 0.1224)			*
						WT on:0.03622±0.002881	WT on:0.03061(0.02041, 0.05102)			
						WT off:0.1028±0.005282	WT off:0.09184(0.07143, 0.1224)			*
						WT on:0.05918±0.003259	WT on:0.06122(0.03316, 0.07908)			
						WT off:0.101±0.005539	WT off:0.09184(0.07143, 0.1224)			*
						WT on:0.06645±0.002859	WT on:0.06633(0.05102, 0.08163)			
						WT off:0.1037±0.005956	WT off:0.09184(0.06888, 0.1327)			*
						WT on:0.07577±0.004314	WT on:0.07143(0.04082, 0.102)			
						WT off:0.1049±0.00573	WT off:0.09184(0.07143, 0.1224)			*
						WT on:0.07755±0.003089	WT on:0.07143(0.06122, 0.09949)			
						WT off:0.08016±0.004771	WT off:0.07143(0.05102, 0.102)			*
						WT on:0.05702±0.002691	WT on:0.05102(0.04082, 0.07143)			
						WT off:0.09807±0.005745	WT off:0.09184(0.06122, 0.1224)			*
						WT on:0.06735±0.003121	WT on:0.06633(0.05102, 0.09184)			
						WT off:0.1003±0.006498	WT off:0.08163(0.05102, 0.1429)			*
						WT on:0.06811±0.004122	WT on:0.06122(0.04082, 0.09184)			
						WT off:0.09773±0.005091	WT off:0.09184(0.06122, 0.1224)			*
						WT on:0.07513±0.003378	WT on:0.07143(0.05102, 0.09184)			
						WT off:0.1142±0.005318	WT off:0.1122(0.08163, 0.1327)			*
						WT on:0.0889±0.003604	WT on:0.09184(0.06378, 0.1122)			
						WT off:0.1018±0.005532	WT off:0.08163(0.06122, 0.1327)			*
						WT on:0.07526±0.003596	WT on:0.07143(0.05102, 0.09949)			
						WT off:0.1087±0.00543	WT off:0.102(0.07143, 0.1327)			*
						WT on:0.08265±0.00336	WT on:0.08163(0.06122, 0.102)			
						WT off:0.1066±0.005084	WT off:0.09184(0.07143, 0.1224)			*
						WT on:0.08431±0.003009	WT on:0.09184(0.06122, 0.102)			
						WT off:0.08639±0.005309	WT off:0.07143(0.05102, 0.1122)			*
						WT on:0.06301±0.003424	WT on:0.06122(0.04082, 0.08163)			
						WT off:0.09263±0.005297	WT off:0.08163(0.06122, 0.1122)			*
						WT on:0.07334±0.003118	WT on:0.07143(0.06122, 0.09184)			
						WT off:0.1033±0.006015	WT off:0.08163(0.06122, 0.1327)			*
						WT on:0.07003±0.004325	WT on:0.06122(0.04082, 0.09184)			
						WT off:0.1008±0.006169	WT off:0.09184(0.06122, 0.1224)			*
						WT on:0.07321±0.003697	WT on:0.07143(0.05102, 0.09184)			
						WT off:0.0941±0.005232	WT off:0.08163(0.06122, 0.1122)			*
						WT on:0.07092±0.003361	WT on:0.06633(0.05102, 0.08163)			
	slice electrophysiology	siEPSC	F	>12wks	KO off: 17 neurons / 6mice<br>KO on: 17 neurons / 6mice	KO off:0.0988±0.002377	KO off:0.102(0.08163, 0.1224)	Two-way repeated measures of ANOVA with Tukey's test	Interaction F(2,7)=0.467, p=0.858<br>light F(2,32)=0.636, p=0.431<br>Hz F(2,7)=886, p=<0.001	ns
						KO on:0.09238±0.002846	KO on:0.09184(0.06122, 0.1224)			
						KO off:0.0949±0.002351	KO off:0.09184(0.07143, 0.1122)			*
						KO on:0.07161±0.002516	KO on:0.07143(0.05102, 0.09184)			
						KO off:0.08547±0.002362	KO off:0.08163(0.06122, 0.102)			*
						KO on:0.06597±0.002488	KO on:0.06122(0.04082, 0.09184)			
						KO off:0.08848±0.002275	KO off:0.09184(0.07143, 0.102)			*
						KO on:0.08049±0.002494	KO on:0.08163(0.06122, 0.102)			
						KO off:0.08439±0.002087	KO off:0.08163(0.06122, 0.102)			ns
						KO on:0.08295±0.002487	KO on:0.08163(0.06122, 0.102)			
						KO off:0.09328±0.002803	KO off:0.08163(0.07143, 0.1122)			ns
						KO on:0.08649±0.002931	KO on:0.08163(0.06122, 0.102)			
						KO off:0.08313±0.002414	KO off:0.08163(0.06122, 0.102)			ns
						KO on:0.08703±0.002616	KO on:0.08163(0.06122, 0.1122)			
						KO off:0.07197±0.002081	KO off:0.07143(0.05102, 0.09184)			ns
						KO on:0.06765±0.002069	KO on:0.06122(0.05102, 0.08163)			
						KO off:0.08223±0.002471	KO off:0.08163(0.06122, 0.102)			ns
						KO on:0.08049±0.002394	KO on:0.08163(0.06122, 0.102)			
						KO off:0.08475±0.003264	KO off:0.08163(0.05102, 0.1122)			ns
						KO on:0.08151±0.003285	KO on:0.07143(0.05102, 0.102)			
						KO off:0.09064±0.002511	KO off:0.09184(0.06122, 0.1122)			ns
						KO on:0.08818±0.002667	KO on:0.09184(0.06122, 0.1122)			
						KO off:0.1035±0.002449	KO off:0.102(0.08163, 0.1327)			ns
						KO on:0.09874±0.002674	KO on:0.102(0.07908, 0.1224)			
						KO off:0.08643±0.002604	KO off:0.08163(0.06122, 0.1122)			ns
						KO on:0.08595±0.002527	KO on:0.08163(0.06122, 0.1122)			
						KO off:0.09622±0.002365	KO off:0.09184(0.07143, 0.1122)			ns
						KO on:0.09322±0.002478	KO on:0.09184(0.07143, 0.1122)			
						KO off:0.09268±0.002112	KO off:0.09184(0.07908, 0.1122)			ns
						KO on:0.08986±0.002095	KO on:0.09184(0.07143, 0.1046)			
						KO off:0.07587±0.00246	KO off:0.07143(0.05102, 0.09184)			ns
						KO on:0.07077±0.002619	KO on:0.06122(0.05102, 0.09184)			
						KO off:0.08361±0.002366	KO off:0.08163(0.06122, 0.102)			ns
						KO on:0.08211±0.002578	KO on:0.08163(0.05102, 0.1122)			
						KO off:0.09136±0.003054	KO off:0.09184(0.06122, 0.1122)			ns
						KO on:0.08355±0.003317	KO on:0.08163(0.05102, 0.1122)			
						KO off:0.08451±0.002384	KO off:0.08163(0.06888, 0.102)			ns
						KO on:0.08487±0.002779	KO on:0.09184(0.06122, 0.1122)			
						KO off:0.08247±0.002916	KO off:0.08163(0.05102, 0.102)			ns
						KO on:0.07953±0.002646	KO on:0.07143(0.05102, 0.102)			
**Figure 5a**	slice electrophysiology	Stimulated PV ratio diagram 10Hz	M	12wks	WT : 504 epochs / 9mice<br>KO : 372 epochs/ 12mice	Chi-square, df	11.76, 2	Chi-square two-sided	p=0.0028	**
		Stimulated PV optoSTIM 10Hz (Mean firing frequency)	M	12wks	WT : 42 neurons / 9mice<br>KO : 31 neurons / 12mice	11.29 ± 1.177	10(9.167, 13.54)	Mann Whitney test, two-tailed	p=0.2561	ns
						13.12 ± 1.292	10(10.00, 19.17)			
**Figure 5b**	slice electrophysiology	Neighboring PV ratio diagram 10Hz	M	12wks	WT : 504 epochs/ 10mice<br>KO : 456 epochs/ 15mice	Chi-square, df	185.4, 2	Chi-square two-sided	p<0.0001	****
		Neighboring PV optoSTIM 10Hz (Mean firing frequency)	M	12wks	WT : 47 neurons / 10mice<br>KO : 42 neurons / 15mice	4.202 ± 0.9015	0(0.00, 10.00)	Mann Whitney, two-tailed	p<0.0001	****
						11.98 ± 1.491	10(6.667, 16.88)			
**Figure 5c**	slice electrophysiology	Stimulated PV ratio diagram 40Hz	M	12wks	WT : 1536 epochs / 8mice<br>KO : 1440 epochs/ 10mice	Chi-square, df	0.5690, 2	Chi-square two-sided	p=0.7524	ns
		Stimulated PV optoSTIM 40Hz (Mean firing frequency)	M	12wks	WT : 32 neurons / 8mice<br>KO : 30 neurons / 10mice	24.14 ± 3.304	32.08(1.667, 40.00)	Mann Whitney test, two-tailed	p=0.671	ns
						25.03± 3.413	33.75(2.292, 40.83)			
**Figure 5d**	slice electrophysiology	Neighboring PV ratio diagram 40Hz	M	12wks	WT : 1776 epochs/ 9mice<br>KO : 816 epochs/ 9mice	Chi-square, df	305.0, 2	Chi-square two-sided	p<0.0001	****
		Neighboring PV optoSTIM 40Hz (Mean firing frequency)	M	12wks	WT : 37 neurons / 9mice<br>KO : 17 neurons / 9mice	5.563 ± 1.982	0(0.00, 2.083)	Mann Whitney, two-tailed	p=0.0067	**
						18.33 ± 4.803	4.167(0.00, 40.42)			
**Figure 6b**	Slice electrophysiology with optogenetic stimulation	10Hz optoSTIM Neighboring PV Ratio diagram	M	12wks	WT : 348 epochs / 13mice<br>WT+MQ : 348 epochs / 13mice	Chi-square, df	WT : 129.0 (195.0, 24.0)<br>WT+MQ : 78 (248, 22)	Chi-square two-sided	p<0.0001	****
		10Hz optoSTIM Neighboring PV Ratio diagram	M	12wks	KO : 300 epochs / 13mice<br>KO+MQ : 300 epochs/ 13mice	Chi-square, df	KO : 104 (148, 48)<br>KO+MQ : 37 (261, 2)	Chi-square two-sided	p<0.0001	****
		10Hz optoSTIM Neighboring PV Firing rate	M	12wks	WT : 29 neurons / 13 mice<br>WT + MQ : 29 neurons / 13 mice<br>KO : 40 neurons / 13 mice<br>KO + MQ : 40 neurons / 13 mice	WT : 7.935±1.172<br>WT+Mef : 4.276±1.222<br>KO : 10.093±1.108<br>KO+Mef : 1.617±0.605	WT : 10.890 (10.903, 0)<br>WT+Mef : 0 (10.883, 0)<br>KO : 10.885 (13.760, 3.542)<br>KO+Mef : 0 (0, 0)	Two-way repeated measures of ANOVA with Šídák's multiple comparisons test	Interaction F (1, 67) = 8.368 p=0.0051<br>Drug F (1, 67) = 53.09 p<0.0001<br>Group F (1, 67) = 0.04349 p=0.8354	Veh-Mef<br>WT : * (p=0.0105)<br>KO : **** (p<0.0001)
**Figure 7a**	slice electrophysiology	NMDA to AMPA ratio	M	12wks	WT : 19 neurons / 3 mice	1.320 ± 0.2620	0.9141 (0.6715, 1.908)	Mann Whitney test, two-tailed	p=0.0125	*
					KO : 13 neurons / 2 mice	0.5506 ± 0.08843	0.5625 (0.2638, 0.7828)			
**figure 7b**	Slice electrophysiology	Pv neuron mEPSC Frequency	M	12wks	WT : 14 neurons / 4mice<br>KO : 13 neurons / 4mice	WT: 6.208 ± 1.080	5.267 (2.825, 8.463)	Unpaired t test, two-tailed	p=0.6140	ns
						KO: 6.862 ± 0.6865	6.767 (5.254, 9.458)			
		Pv neuron mEPSC Amplitude	M	12wks	WT : 14 neurons / 4mice<br>KO : 13 neurons / 4mice	WT: 12.96 ± 0.8069	12.02 (10.40, 15.31)	Unpaired t test, two-tailed	p=0.5114	ns
						KO: 14.96 ± 1.474	12.61 (10.60, 21.23)			
**figure 7c**	Slice electrophysiology with optogenetic stimulation	10Hz optoSTIM Neighboring PV Ratio diagram	M	12wks	WT : 516 epochs / 18mice<br>WT+DCS : 516 epochs/ 18mice	Chi-square, df	WT : 248 (255, 21)<br>WT+DCS : 178 (251, 87)	Chi-square two-sided	p<0.0001	****
		10Hz optoSTIM Neighboring PV Ratio diagram	M	12wks	KO : 408 epochs / 19mice<br>KO+DCS : 408 epochs/ 19mice	Chi-square, df	KO : 117 (214, 77)<br>KO+DCS : 137 (193, 78)	Chi-square two-sided	p<0.0001	****
		10Hz optoSTIM Neighboring PV Firing rate	M	12wks	WT : 43 neurons / 18 mice<br>WT + DCS : 43 neurons / 18 mice<br>KO : 38 neurons / 19 mice<br>KO + DCS : 38 neurons / 19 mice	WT : 6.693 ± 0.993<br>WT+DCS : 8.692 ± 1.402<br>KO : 13.015 ± 1.743<br>KO+DCS: 18.158 ± 2.174	WT : 8.970 (10.894, 0)<br>WT+DCS : 10.042 (16.155, 0)<br>KO : 10.892 (17.053, 10.431)<br>KO+DCS: 15.434 (21.659, 10.893)	Two-way repeated measures of ANOVA with Šídák's multiple comparisons test	Interaction F (1, 79) = 3.494 p=0.0653<br>Drug F (1, 79) = 18.04 p<0.0001<br>Group F (1, 79) = 14.19 p=0.0003	Veh-DCS<br>WT : ns (p=0.1654)<br>KO : *** (p=0.0001)

**figure 7d**	Slice electrophysiology with optogenetic stimulation	10Hz optoSTIM<br>Neighboring PV<br>Ratio diagram	M	12wks	WT+DCS+Veh : 192 epochs / 10mice<br><br>WT+DCS+Mef : 240 epochs / 4mice	Chi-square, df	WT+DCS+Veh :<br>ACSF : 65 (121, 6)<br>DCS :54 (103, 35)<br>Veh : 49 (109, 34)<br>WT+DCS+Mef :<br>ACSF : 90 (232, 2)<br>DCS : 67 (229, 28)<br>Mef : 12 (312, 0)	Chi-square<br>two-sided	WT+DCS+Veh :<br>WT vs. DCS, p<0.0001<br>DCS vs. Veh, p=0.8077<br><br>WT+DCS+Mef :<br>WT vs. DCS, p<0.0001<br>DCS vs. Mef, p<0.0001	WT+DCS+Veh :<br>WT vs. DCS, ****<br>DCS vs. Veh, ns<br><br>WT+DCS+Mef :<br>WT vs. DCS, ****<br>DCS vs. Mef, ****
		10Hz optoSTIM<br>Neighboring PV<br>Ratio diagram	M	12wks	KO+DCS+Veh : 252 epochs / 12mice<br><br>KO+DCS+Mef : 240 epochs / 12mice	Chi-square, df	KO+DCS+Veh :<br>ACSF : 98 (114, 40)<br>DCS :91 (95, 66)<br>Veh : 82 (150, 20)<br>KO+DCS+Mef :<br>ACSF : 55 (147, 38)<br>DCS : 70 (141, 29)<br>Mef : 9 (279, 0)	Chi-square<br>two-sided	KO+DCS+Veh :<br>KO vs. DCS, p<0.0001<br>DCS vs. Veh, p<0.0001<br><br>KO+DCS+Mef :<br>KO vs. DCS, p=0.0001<br>DCS vs. Mef, p<0.0001	KO+DCS+Veh :<br>KO vs. DCS, ****<br>DCS vs. Veh, ****<br><br>WT+DCS+Mef :<br>KO vs. DCS, ****<br>DCS vs. Mef, ****
		10Hz optoSTIM<br>Neighboring PV<br>Firing rate	M	12wks	WT+DCS+Veh : 16 neurons / 10 mice<br>WT+DCS+Mef : 27 neurons / 10 mice<br><br>KO+DCS+Veh : 21 neurons / 12 mice<br>KO+DCS+Mef: 20 neurons / 12 mice	WT+DCS+Veh :<br>WT : 5.658 ± 1.544<br>DCS : 8.236 ± 2.568<br>Veh : 6.889 ± 2.285<br><br>WT+DCS+Mef :<br>WT : 3.247 ± 0.942<br>DCS : 5.244 ± 1.714<br>Mef : 1.763 ± 1.763<br><br>KO+DCS+Veh :<br>KO : 8.233 ± 1.834<br>DCS : 15.412 ± 3.371<br>Veh : 14.710 ± 4.201<br><br>KO+DCS+Mef :<br>KO : 10.067 ± 1.340<br>DCS : 14.378 ± 1.615<br>Mef : 0.408 ± 0.408	WT+DCS+Veh :<br>WT : 4.485 (10.883, 0)<br>DCS : 4.364 (10.976, 0)<br>Veh : 0 (10.887, 0)<br>WT+DCS+Mef :<br>WT : 0 (8.975, 0)<br>DCS : 0 (10.892, 0)<br>Mef : 0 (0, 0)<br>KO+DCS+Veh :<br>KO : 10.886 (12.254, 0)<br>DCS : 11.802 (22.067, 0)<br>Veh : 0 (22.981, 0)<br>KO+DCS+Mef :<br>KO : 10.892 (12.482, 8.488)<br>DCS : 11.797 (20.994, 10.897)<br>Mef : 0 (0, 0)	Two-way repeated measures of ANOVA with Šídák's multiple comparisons test	WT<br>Interaction F (2, 82) = 0.9416 p=0.3942<br>Session F (1.815, 74.42) = 3.401 p=0.0430<br>Treatment F (1, 41) = 2.347 p=0.1332<br><br>KO<br>Interaction F (2, 78) = 17.66 p<0.0001<br>Session F (1.544, 60.21) = 14.20 p<0.0001<br>Treatment F (1, 39) = 2.026 p=0.1626	WT :<br>Veh vs. Mef, ns (p=0.2346)<br>KO :<br>Veh vs. Mef, ** (p=0.0086)
**figure 8b**	Three chamber test with optogenetic stimulation	Shank2 WT PV-cre; AAV-EYFP	M	>12wks	n=15	Social<br>pre:190.4±14.91<br>opto:154.7±19.12<br>post:183±27.17<br>Object<br>pre:43.01±6.755<br>opto:45.49±6.941<br>post:39.56±9.08	Social<br>pre:196.6(138, 221.2)<br>opto:157.3(83.28, 191.6)<br>post:171.1(79.74, 262.9)<br>Object<br>pre:39.37(22.89, 64.03)<br>opto:37.97(24.99, 68.57)<br>post:36.7(11.41, 64.53)	Two-way repeated measures of ANOVA with Tukey's test	Interaction F(2,56)=1.353, p=0.2667<br>light F(2,56)=0.8813, p=0.4199<br>target F(1,28)=62.58, p<0.0001	
		Shank2 KO PV-cre; AAV-EYFP			n=11	Social<br>pre:109.5±15.92<br>opto:99.44±17.12<br>post:86.04±16.75<br>Object<br>pre:27.16±3.323<br>opto:36.26±6.741<br>post:36.98±4.837	Social<br>pre:105.3(55.45, 159.8)<br>opto:110.9(44.64, 140.2)<br>post:79.91(61.36, 113.7)<br>Object<br>pre:24.69(19.59, 34.1)<br>opto:29(23.16, 47.15)<br>post:35.23(24.72, 49.75)	Two-way repeated measures of ANOVA with Tukey's test	Interaction F(2,10)=1.296, p=0.2849<br>light F(2,40)=0.2686, p=0.7658<br>target F(1,20)=26.45, p<0.0001	
**figure 8c**	Three chamber test with optogenetic stimulation	Shank2 WT PV-cre; AAV-ChR2	M	>12wks	n=20	Social<br>pre:210.4±12<br>opto:233.7±15.99<br>post:193.3±11.69<br>Object<br>pre:44.23±5.975<br>opto:44.83±7.566<br>post:52.31±6.427	Social<br>pre:202.4(181.9, 253)<br>opto:246.2(162.3, 297.6)<br>post:193(165.6, 221.6)<br>Object<br>pre:32.5(24.91, 65.45)<br>opto:35.89(25.84, 62.68)<br>post:50.4(32.03, 65.91)	Two-way repeated measures of ANOVA with Tukey's test	Interaction F(2,76)=4.098, p=0.0204<br>light F(2,76)=2.069, p=0.1334<br>target F(1,38)=210, p<0.0001	
		Shank2 KO PV-cre; AAV-ChR2			n=16	Social<br>pre:101.8±10.91<br>opto:161.6±21.33<br>post:116±14.67<br>Object<br>pre:33.73±4.116<br>opto:46.13±6.356<br>post:39.4±7.005	Social<br>pre:105.6(60.54, 137.6)<br>opto:140.4(91.31, 254.1)<br>post:95.48(72.08, 162.8)<br>Object<br>pre:32.55(19.64, 43.6)<br>opto:46.21(30.95, 65.71)<br>post:42.51(12.18, 59.84)	Two-way repeated measures of ANOVA with Tukey's test	Interaction F(2,60)=2.649, p=0.0790<br>light F(2,60)=5.775, p=0.0051<br>target F(1,30)=54.41, p<0.0001	
**figure 8d**	Three chamber test with optogenetic stimulation	Shank2 WT PV-cre; AAV-ChETA	M	>12wks	n=8	Social<br>pre:174.9±24.44<br>opto:190.3±19.04<br>post:173.3±23.92<br>Object<br>pre:40.66±8.005<br>opto:46.92±9.674<br>post:37.42±7.723	Social<br>pre:197.2(161.5, 216.2)<br>pre:198.8(177.6, 226.7)<br>pre:165.6(130.8, 197)<br>Object<br>pre:52.82(18.48, 57.22)<br>pre:49.26(29.07, 69.28)<br>pre:43.33(14.5, 48.96)	Two-way repeated measures of ANOVA with Tukey's test	Interaction F(2,28)=0.0775, p=0.9256<br>light F(2,28)=0.643, p=0.5333<br>target F(1,14)=50.6, p<0.0001	
		Shank2 KO PV-cre; AAV-ChETA			n=10	Social<br>pre:133.6±19.47<br>opto:96.63±10.24<br>post:97.32±20.75<br>Object<br>pre:39.11±5.721<br>opto:34.21±4.609<br>post:36.01±5.835	Social<br>pre:137.3(73.69, 192.6)<br>pre:106.2(71.8, 125.6)<br>pre:64.55(55.95, 143.4)<br>Object<br>pre:38.9(24.07, 52.23)<br>pre:34.13(20.05, 44.59)<br>pre:33.85(21.87, 49.74)	Two-way repeated measures of ANOVA with Tukey's test	Interaction F(2,36)=1.653, p=0.2057<br>light F(2,36)=2.568, p=0.0907<br>target F(1,18)=27.73, p<0.0001	
**figure 8e**	Three chamber test with optogenetic stimulation	Shank2 WT PV-cre; AAV-SSFO	M	>12wks	n=15	Social<br>pre:172±11.55<br>opto:172.7±24.37<br>post:178.1±24.02<br>Object<br>pre:79.63±7.78<br>opto:94.47±16.69<br>post:79.4±15.6	Social<br>pre:164.1(137.8, 196.9)<br>pre:177.2(70.47, 243.4)<br>pre:166.7(107.6, 233)<br>Object<br>pre:79.18(55.45, 97.42)<br>pre:89.22(69.4, 118.8)<br>pre:56.86(30.83, 148.1)	Two-way repeated measures of ANOVA with Tukey's test	Interaction F(2,56)=0.2196, p=0.7<br>light F(2,56)=0.122, p=0.8851<br>target F(1,28)=27.31, p<0.0001	
		Shank2 KO PV-cre; AAV-SSFO			n=14	Social<br>pre:88.48±9.674<br>opto:93.56±10.94<br>post:81.89±15.69<br>Object<br>pre:37.21±4.903<br>opto:44.05±8.782<br>post:44.11±5.344	Social<br>pre:80.31(61.69, 117.7)<br>pre:84.12(56.14, 128.1)<br>pre:55.81(34.62, 133.6)<br>Object<br>pre:37.19(22.1, 48.12)<br>pre:29.25(18.41, 58.14)<br>pre:41.07(28.45, 54.12)	Two-way repeated measures of ANOVA with Tukey's test	Interaction F(2,54)=0.333, p=0.7180<br>light F(2,54)=0.377, p=0.688<br>target F(1,28)=23.41, p<0.0001	
**Figure 8h**	in vivo recording with optogenetic stimulation Shank2 **KO** PV-cre; AAV-EYFP Shank2 **KO** PV-cre; AAV-ChR2	Target	M	>12wks	EYFP=163 neurons/4 mice<br>ChR2=364 neurons/9 mice	EYFP nT n=68,T 95<br>ChR2 nT n=181, T 183		Chi-square test<br>two-sided	p=0.0691	ns
		Side			EYFP=163 neurons/4 mice<br>ChR2=364 neurons/9 mice	EYFP nSi n=121,Si 42<br>ChR2 nSi n=296, Si 68		Chi-square test<br>two-sided	p=0.0643	ns
		S-sepecific			EYFP=163 neurons/4 mice<br>ChR2=364 neurons/9 mice	EYFP nS-spc n=150, S-spc 13<br>ChR2 nS-spc n=339, S-spc 25		Chi-square test<br>two-sided	p=0.786	ns
		O-sepecific			EYFP=163 neurons/4 mice<br>ChR2=364 neurons/9 mice	EYFP nO-spc n=148, O-spc 15<br>ChR2 nO-spc n=347, O-spc 17		Chi-square test<br>two-sided	p=0.0441	*
		E-sepecific			EYFP=163 neurons/4 mice<br>ChR2=364 neurons/9 mice	EYFP nE-spc n=118, E-spc 45<br>ChR2 nE-spc n=276, E-spc 88		Chi-square test<br>two-sided	p=0.702	ns
	in vivo recording with optogenetic stimulation Shank2 **WT** PV-cre; AAV-EYFP Shank2 **WT** PV-cre; AAV-ChR2	Target	M	>12wks	EYFP=194 neurons/7 mice<br>ChR2=72 neurons/8 mice	EYFP nT n=113,T 85<br>ChR2 nT n=28, T 44		Chi-square test<br>two-sided	p=0.552	ns
		Side			EYFP=194 neurons/7 mice<br>ChR2=72 neurons/8 mice	EYFP nSi n=154,Si 44<br>ChR2 nSi n=26, Si 46		Chi-square test<br>two-sided	p=0.0213	*
		S-sepecific			EYFP=194 neurons/7 mice<br>ChR2=72 neurons/8 mice	EYFP nS-spc n=184, S-spc 14<br>ChR2 nS-spc n=60, S-spc 12		Chi-square test<br>two-sided	p=0.0181	*
		O-sepecific			EYFP=194 neurons/7 mice<br>ChR2=72 neurons/8 mice	EYFP nO-spc n=189 O-spc 9<br>ChR2 nO-spc n=69, O-spc 3		Chi-square test<br>two-sided	p=0.894	ns
		E-sepecific			EYFP=194 neurons/7 mice<br>ChR2=72 neurons/8 mice	EYFP nE-spc n=173, E-spc 25<br>ChR2 nE-spc n=64, E-spc 8		Chi-square test<br>two-sided	p=0.737	ns
**Figure 8i**	in vivo recording with optogenetic stimulation	Shank2 KO PV-cre; AAV-EYFP	M	>12wks	n=163/4			Chi-square test<br>two-sided	p=0.0284	*
		Shank2 KO PV-cre; AAV-ChR2			n=364/9					
**figure 9b**	in vivo recording with optogenetic stimulation	Theta	M	>12wks	WT EYFP<br>n=197;<br>WT ChR2<br>n=63;<br>KO EYFP<br>n=106;<br>KO ChR2<br>n=259		WT-EYFP 0.1402(0.09153,0.2015)<br>WT-ChR2 0.1357(0.08129,0.8413)<br>KO-EYFP 0.1430(0.10000,0.2451)<br>KO-ChR2 0.1348(0.08531,0.1752)	Two-way repeated measures of ANOVA with Tukey's test	Interaction F(1,760)=12.99,p=0.0003;<br>Genotype F(2,760)=1.92, p=0.17;<br>virus F(2,760)=0.15, p=0.7;	
		Alpha					WT-EYFP 0.1313(0.07711,0.1829)<br>WT-ChR2 0.1248(0.06955,0.2508)<br>KO-EYFP 0.1279(0.1006,0.2245)<br>KO-ChR2 0.1184(0.07079,0.1645)	Two-way repeated measures of ANOVA with Tukey's test	Interaction F(1,760)=23.11, p=0.0000;<br>Genotype F(2, 760)=3.3, p=0.096;<br>virus F(2,760)=0.01, p=0.939	
		Low gamma					WT-EYFP 0.05632(0.03350,0.08824)<br>WT-ChR2 0.07110(0.04168,0.1219)<br>KO-EYFP0.06112(0.04176,0.09576)<br>KO-ChR2 0.04520(0.02399,0.07196)	Two-way repeated measures of ANOVA with Tukey's test	Interaction F(1,760)=38.13, p=0.0000;<br>Genotype F(2, 760)=3.3, p=0.096;<br>virus F(2,760)=0.01, p=0.939	
		High gamma					WT-EYFP 0.09829(0.05201,0.1450)<br>WT-ChR2 0.09650(0.05480,0.1965)<br>KO-EYFP 0.09487(0.06108,0.1565)<br>KO-ChR2 0.06220(0.03775,0.09909)	Two-way repeated measures of ANOVA with Tukey's test	Interaction F(1,760)=30.08, p=0.000;<br>Genotype F(2,760)=14.92, p=0.001;<br>virus F(2,760)=5.04, p=0.025	
**figure 9c**	Three chamber test with MQ local infusion	WT_Veh sniffing time	M	>12wks	n=11	Social: 169.9±19.87<br>Object: 48.66±9.045	Social: 163.4 (124.1, 225.6)<br>Object: 39.74 (32.03, 54.08)	Two-way repeated measures of ANOVA	Interaction F (1, 10) = 0.1640, p=0.6940;<br>Treatment F (1, 10) = 1.384, p=0.2667;<br>Target F (1, 10) = 46.88, p<0.0001	
		WT_MQ sniffing time				Social: 154.8±16.5<br>Object: 40.99±8.362	Social: 156.7 (118.6, 201.2)<br>Object: 32.78 (20.19, 71.6)			
		KO_Veh sniffing time			n=8	Social: 115.8±15.57<br>Object: 41.38±8.135	Social: 105.7 (73.31, 166.3)<br>Object: 37.39 (20.28, 64.65)	Two-way repeated measures of ANOVA	Interaction F (1, 7) = 0.5622, p=0.4778;<br>Treatment F (1, 7) = 1.490, p=0.2617;<br>Target F (1, 7) = 50.58, p=0.0002	
		KO_MQ sniffing time				Social: 137.5±14.23<br>Object: 48.83±7.575	Social: 144.2 (96.12, 173.7)<br>Object: 46.93 (29.01, 59.01)			
		WT preference index			n=11	Vehicle: 0.5425±0.0696<br>MQ: 0.5852±0.05852	Vehicle: 0.6253 (0.4025, 0.7057)<br>MQ: 0.4539 (0.4119, 0.7527)	Two-way repeated measures of ANOVA	Interaction F (1, 17) = 0.1441, p=0.7089;<br>Genotype F (1, 17) = 1.327, p=0.2653;<br>Treatment F (1, 17) = 0.1205, p=0.7327	
		KO preference index			n=8	Vehicle: 0.4775±0.07985<br>MQ: 0.4756±0.05706	Vehicle: 0.5291 (0.2302, 0.6575)<br>MQ: 0.4903 (0.3223, 0.5709)			

**figure 9d**	Three chamber test with Connexin-36 knockdown	shRNA1 Control sniffing time	M	>12wks	n=8	Social: 206.8±21.52	Social: 230.4 (137.3, 259.3)	Two-way repeated measures of ANOVA	Interaction F (1, 15) = 1.541, p=0.2335;<br>Treatment F (1, 15) = 0.8851, p=0.3617;<br>Target F (1, 15) = 130.4, p<0.0001	
						Object: 45.96±2.649	Object: 46.71 (41.46, 51.91)			
		shRNA1 Knockdown sniffing time			n=9	Social: 241±20.83	Social: 245.7 (200, 273.6)			
						Object: 40.95±8.143	Object: 38.94 (24.99, 47.46)			
		shRNA1 Preference index			Control: n=8<br>Knockdown: n=9	Control: 0.6229±0.02606	Control: 0.6474 (0.5708, 0.6779)	Mann Whitney test, two-tailed	p=0.0274	*
						Knockdown: 0.704±0.05778	Knockdown: 0.698 (0.6749, 0.8334)			
		shRNA2 Control sniffing time		>10wks	n=10	Social: 199.2±14.06	Social: 192.5 (165.9, 226.9)	Two-way repeated measures of ANOVA	Interaction F (1, 18) = 2.577, p=0.1258;<br>Treatment F (1, 18) = 5.528, p=0.0303;<br>Target F (1, 18) = 159.7, p<0.0001	
						Object: 56.96±6.786	Object: 53.58 (41.12, 73.93)			
		shRNA2 Knockdown sniffing time			n=10	Social: 241±12.63	Social: 240.2 (208.1, 266.7)			
						Object: 57.27±9.442	Object: 48.79 (38.59, 81.93)			
		shRNA2 Preference index			Control: n=10<br>Knockdown: n=10	Control: 0.5491±0.0514	Control: 0.5426 (0.4253, 0.7151)	Unpaired t test, two-tailed	p=0.3869	ns
						Knockdown: 0.6176±0.05773	Knockdown: 0.6486 (0.4757, 0.7476)			

Figure	Assay Performed	Paremeter	Comparison WT vs KO			Descriptive Statistics		Statistical Analysis		
			Sex	Age	n	Average ± SEM	Median (2Q, 3Q)	Statistical Test	Significance	Summary
**Supplementary figure1c**	Linear chamber test	1st SO	M	>12wks	n=5	WT_S 189.9±15.37<br>WT_O 80.92±10.11<br>KO_S 131.8±16.63<br>KO_O 106.3±17.97		Two-way repeated measures of ANOVA with Tukey's test	Interaction F(1,112)=11.13, p=0.0012<br>genotyep F(1,112)=1.071, p=0.1054<br>target F(1,112)=2.665, p=0.3030	
		2nd SO			n=5	WT_S 137.4±16.64<br>WT_O 123.4±15.54<br>KO_S 145.7±16.45<br>KO_O 176.9±23.07		Two-way repeated measures of ANOVA with Tukey's test	Interaction F(1,112)=0.0254, p=0.8737<br>genotyep F(1,112)=1.787, p=0.1840<br>target F(1,112)=0.83, p=0.3632	
**Supplementary figure1d**	Linear chamber test	WT	M	>12wks	n=5	E-E 0.6384±0.03124<br>1stSO 0.933±0.07769<br>2ndSO 0.8017±0.05079		Friedman test with Dunn's test<br>p=0.0277,<br>two-tailed	E_E vs. 1stSO p<0.0001<br>E_E vs. 2ndSO p=0.0287<br>1stSO vs. 2ndSO p=0.3160	
		KO			n=5	E-E 0.4822±0.02585<br>1stSO 0.75±0.04547<br>2ndSO 0.5671±0.02623		Friedman test with Dunn's test<br>p=0.0005<br>two-tailed	E_E vs. 1stSO p=0.0246<br>E_E vs. 2ndSO p=0.2710<br>1stSO vs. 2ndSO p>0.999	
**Supplimentary figure 3c**	Linear Chamber	Relative burst firing in plnh neurons burst criteria: < 6 ms	M	12wks	WT neuron n=36 / mice n=5	E-R: 0.003262±0.002016	E-R: 0.001701 (-0.005057, 0.01093)	Two-way repeated measures of ANOVA with Tukey's test	interaction p=0.1475<br>target p=0.0105<br>genotype p=0.1255	WT<br>E-R vs. O-R: p=0.7282<br>E-R vs. S-R: p=0.0099<br>O-R vs. S-R: p=0.0738<br>KO<br>E-R vs. O-R: p=0.3629<br>E-R vs. S-R: p=0.9071<br>O-R vs. S-R: p=0.1785
						O-R: 0.005101±0.002033	O-R: 0.002666 (-0.002752, 0.01087)			
						S-R: 0.01044±0.004012	S-R: 0.002847 (-0.004043, 0.01902)			
					KO neuron n=40 / mice n=5	E-R: 0.001405±0.003525	E-R: -0.0003666 (-0.007087, 0.001527)			
						O-R: -0.001725±0.002523	O-R: -0.001252 (-0.008967, 0.001296)			
						S-R: 0.002371±0.002567	S-R: -0.00112 (-0.005693, 0.003843)			
		Relative burst firing in plnh neurons burst criteria: < 12 ms	M	12wks	WT neuron n=36 / mice n=5	E-R: 0.01394±0.007035	E-R: 0.007572 (-0.01925, 0.0501)	Two-way repeated measures of ANOVA with Tukey's test	interaction p=0.285<br>target p=0.0036<br>genotype p=0.0094	WT<br>E-R vs. O-R: p=0.892<br>E-R vs. S-R: p=0.0253<br>O-R vs. S-R: p=0.0068<br>KO<br>E-R vs. O-R: p=0.5353<br>E-R vs. S-R: p=0.8501<br>O-R vs. S-R: p=0.244
						O-R: 0.0112±0.0094	O-R: 0.01508 (-0.02136, 0.04467)			
						S-R: 0.02974±0.009894	S-R: 0.01848 (-0.003129, 0.05393)			
					KO neuron n=40 / mice n=5	E-R: -0.006337±0.006772	E-R: -0.009613 (-0.03813, 0.01945)			
						O-R: -0.01242±0.006205	O-R: -0.01052 (-0.03548, 0.006347)			
						S-R: -0.003243±0.006009	S-R: -0.007794 (-0.02632, 0.02209)			
		Relative burst firing in plnh neurons burst criteria: < 24 ms	M	12wks	WT neuron n=36 / mice n=5	E-R: 0.008062±0.01518	E-R: -0.006956 (-0.04343, 0.07989)	Two-way repeated measures of ANOVA with Tukey's test	interaction p=0.2262<br>target p=0.0211<br>genotype p=0.2902	WT<br>E-R vs. O-R: p=0.9086<br>E-R vs. S-R: p=0.0529<br>O-R vs. S-R: p=0.0177<br>KO<br>E-R vs. O-R: p=0.5131<br>E-R vs. S-R: p=0.9993<br>O-R vs. S-R: p=0.4912
						O-R: 0.00398±0.02002	O-R: 0.009604 (-0.06664, 0.06066)			
						S-R: 0.03101±0.01981	S-R: 0.01564 (-0.04111, 0.09057)			
					KO neuron n=40 / mice n=5	E-R: -0.006695±0.01596	E-R: -0.002201 (-0.06588, 0.03422)			
						O-R: -0.01694±0.01567	O-R: -0.0159 (-0.0915, 0.02187)			
						S-R: -0.006362±0.01565	S-R: -0.01412 (-0.06181, 0.0559)			
		Relative burst firing in plnh neurons	M	12wks	WT neuron n=36 / mice n=5<br>KO neuron n=40 / mice n=5	WT - 3 ms: 0.0008549±0.000584	WT - 3 ms: 0.000162 (-0.0002875, 0.002879)	Two-way ANOVA	genotype p=0.1884<br>target p=0.6032	
						KO - 3 ms: -0.00007958±0.0004166	KO - 3 ms: 0 (-0.0007075, 0.0005673)			
						WT - 6 ms: 0.006266±0.001654	WT - 6 ms: 0.002531 (-0.00358, 0.01242)		genotype p=0.0186<br>target p=0.2212	
						KO - 6 ms: 0.0006843±0.001673	KO - 6 ms: -0.00111 (-0.006468, 0.002366)			
						WT - 9 ms: 0.01144±0.003185	WT - 9 ms: 0.009649 (-0.004543, 0.02518)		genotype p=0<br>target p=0.0689	
						KO - 9 ms: -0.004907±0.002214	KO - 9 ms: -0.00346 (-0.01706, 0.006914)			
						WT - 12 ms: 0.0183±0.005131	WT - 12 ms: 0.01495 (-0.01652, 0.04937)		genotype p=0<br>target p=0.1882	
						KO - 12 ms: -0.007334±0.003645	KO - 12 ms: -0.009655 (-0.03268, 0.01685)			
						WT - 15 ms: 0.02109±0.007274	WT - 15 ms: 0.02371 (-0.02753, 0.07011)		genotype p=0.0008<br>target p=0.3046	
						KO - 15 ms: -0.009249±0.005344	KO - 15 ms: -0.01334 (-0.047, 0.01994)			
						WT - 18 ms: 0.0195±0.008846	WT - 18 ms: 0.005626 (-0.03531, 0.08244)		genotype p=0.004<br>target p=0.3611	
						KO - 18 ms: -0.01217±0.006585	KO - 18 ms: -0.01352 (-0.05908, 0.01864)			
						WT - 21 ms: 0.01738±0.01027	WT - 21 ms: 0.007329 (-0.04588, 0.0923)		genotype p=0.0218<br>target p=0.4719	
						KO - 21 ms: -0.01252±0.008065	KO - 21 ms: -0.01603 (-0.06734, 0.02597)			
						WT - 24 ms: 0.01435±0.01063	WT - 24 ms: 0.004969 (-0.04441, 0.07856)		genotype p=0.081<br>target p=0.5537	
						KO - 24 ms: -0.01±0.009034	KO - 24 ms: -0.01139 (-0.06472, 0.03588)			
						WT - 27 ms: 0.01437±0.01129	WT - 27 ms: 0.007594 (-0.05129, 0.09735)		genotype p=0.1165<br>target p=0.5591	
						KO - 27 ms: -0.009104±0.009791	KO - 27 ms: -0.007335 (-0.06343, 0.03924)			
						WT - 30 ms: 0.01766±0.01161	WT - 30 ms: 0.007636 (-0.04901, 0.09823)		genotype p=0.114<br>target p=0.581	
						KO - 30 ms: -0.007177±0.0105	KO - 30 ms: -0.007375 (-0.06475, 0.04397)			
**Supplimentary figure 3d**	Linear Chamber	Relative burst firing in pExc neurons burst criteria: < 6 ms	M	12wks	WT neuron n=273 / mice n=5	E-R: -0.008936±0.00454	E-R: 0 (-0.008985, 0.00841)	Two-way repeated measures of ANOVA with Tukey's test	interaction p=0.9832<br>target p=0.6376<br>genotype p=0.6945	WT<br>E-R vs. O-R: p=0.9159<br>E-R vs. S-R: p=0.775<br>O-R vs. S-R: p=0.9574<br>KO<br>E-R vs. O-R: p=0.9847<br>E-R vs. S-R: p=0.7882<br>O-R vs. S-R: p=0.8761
						O-R: -0.008103±0.004781	O-R: 0 (-0.009825, 0.008641)			
						S-R: -0.007517±0.00476	S-R: 0 (-0.00971, 0.01104)			
					KO neuron n=316 / mice n=5	E-R: -0.01138±0.005067	E-R: 0 (-0.008615, 0.008966)			
						O-R: -0.01105±0.004913	O-R: 0 (-0.009748, 0.007651)			
						S-R: -0.0101±0.005162	S-R: 0 (-0.006798, 0.008206)			
		Relative burst firing in pExc neurons burst criteria: < 12 ms	M	12wks	WT neuron n=273 / mice n=5	E-R: -0.01726±0.006726	E-R: -0.00118 (-0.03635, 0.02704)	Two-way repeated measures of ANOVA with Tukey's test	interaction p=0.3588<br>target p=0.407<br>genotype p=0.3957	WT<br>E-R vs. O-R: p=0.3441<br>E-R vs. S-R: p=0.1682<br>O-R vs. S-R: p=0.9111<br>KO<br>E-R vs. O-R: p=0.9956<br>E-R vs. S-R: p=0.9988<br>O-R vs. S-R: p=0.999
						O-R: -0.009967±0.007546	O-R: 0 (-0.0291, 0.03182)			
						S-R: -0.007818±0.008909	S-R: -0.00027 (-0.03511, 0.02951)			
					KO neuron n=316 / mice n=5	E-R: -0.01948±0.006394	E-R: -0.002125 (-0.03521, 0.01763)			
						O-R: -0.01992±0.007046	O-R: -0.00171 (-0.04194, 0.02222)			
						S-R: -0.01971±0.006995	S-R: -0.002805 (-0.03089, 0.02666)			
		Relative burst firing in pExc neurons burst criteria: < 24 ms	M	12wks	WT neuron n=273 / mice n=5	E-R: -0.02013±0.008098	E-R: -0.00915 (-0.07154, 0.04804)	Two-way repeated measures of ANOVA with Tukey's test	interaction p=0.6028<br>target p=0.5874<br>genotype p=0.9461	WT<br>E-R vs. O-R: p=0.8989<br>E-R vs. S-R: p=0.6533<br>O-R vs. S-R: p=0.3846<br>KO<br>E-R vs. O-R: p=0.9309<br>E-R vs. S-R: p=0.9497<br>O-R vs. S-R: p=0.9984
						O-R: -0.01749±0.009027	O-R: 0.006398 (-0.07143, 0.05497)			
						S-R: -0.02541±0.00988	S-R: -0.00434 (-0.07485, 0.04827)			
					KO neuron n=316 / mice n=5	E-R: -0.019±0.008008	E-R: -0.007555 (-0.05659, 0.03731)			
						O-R: -0.02101±0.008516	O-R: -0.00833 (-0.06132, 0.04313)			
						S-R: -0.02071±0.008857	S-R: -0.00466 (-0.06015, 0.04266)			
		Relative burst firing in pExc neurons	M	12wks	WT neuron n=273 / mice n=5<br>KO neuron n=316 / mice n=5	WT - 3 ms: -0.0002943±0.0004508	WT - 3 ms: 0 (0, 0.001387)	Two-way ANOVA	genotype p=0.0444<br>target p=0.9909	
						KO - 3 ms: -0.004543±0.001918	KO - 3 ms: 0 (-0.0004425, 0.001401)			
						WT - 6 ms: -0.008186±0.002708	WT - 6 ms: 0 (-0.00942, 0.009107)		genotype p=0.479<br>target p=0.9651	
						KO - 6 ms: -0.01085±0.002912	KO - 6 ms: 0 (-0.008563, 0.008058)			
						WT - 9 ms: -0.01094±0.004104	WT - 9 ms: 0 (-0.02269, 0.01981)		genotype p=0.626<br>target p=0.8121	
						KO - 9 ms: -0.01361±0.003274	KO - 9 ms: -0.00037 (-0.02316, 0.01439)			
						WT - 12 ms: -0.01168±0.004488	WT - 12 ms: 0 (-0.03424, 0.02944)		genotype p=0.1655<br>target p=0.8294	
						KO - 12 ms: -0.0197±0.003932	KO - 12 ms: -0.002025 (-0.03578, 0.02228)			
						WT - 15 ms: -0.01554±0.004697	WT - 15 ms: 0 (-0.05318, 0.03546)		genotype p=0.3469<br>target p=0.985	
						KO - 15 ms: -0.02122±0.004095	KO - 15 ms: -0.004045 (-0.04821, 0.02598)			
						WT - 18 ms: -0.01531±0.004765	WT - 18 ms: 0.001863 (-0.06771, 0.04224)		genotype p=0.6038<br>target p=0.993	
						KO - 18 ms: -0.02099±0.00432	KO - 18 ms: -0.006085 (-0.05356, 0.03277)			
						WT - 21 ms: -0.02141±0.005067	WT - 21 ms: -0.00149 (-0.07003, 0.04508)		genotype p=0.7323<br>target p=0.9665	
						KO - 21 ms: -0.02123±0.00471	KO - 21 ms: -0.005255 (-0.05544, 0.0361)			
						WT - 24 ms: -0.02101±0.005209	WT - 24 ms: -0.00176 (-0.07182, 0.04998)		genotype p=0.6889<br>target p=0.9577	
						KO - 24 ms: -0.02024±0.004884	KO - 24 ms: -0.006165 (-0.05891, 0.04111)			
						WT - 27 ms: -0.02297±0.005531	WT - 27 ms: -0.00124 (-0.07813, 0.05529)		genotype p=0.3809<br>target p=0.9709	
						KO - 27 ms: -0.01845±0.004867	KO - 27 ms: -0.00773 (-0.06171, 0.04231)			
						WT - 30 ms: -0.02386±0.005595	WT - 30 ms: -0.00357 (-0.0826, 0.0612)		genotype p=0.2267<br>target p=0.9589	
						KO - 30 ms: -0.0169±0.004896	KO - 30 ms: -0.00675 (-0.06276, 0.0474)			
**Supplimentary figure 3e**	Linear Chamber	Relative burst firing in plnh neurons burst criteria: < 12 ms	M	12wks	WT neuron n=36 / mice n=5	E-R: -0.03473±0.01392	E-R: -0.02305 (-0.07441, 0.02003)	Two-way repeated measures of ANOVA with Tukey's test	interaction p=0.7427<br>target p=0.0658<br>genotype p=0.3082	WT<br>E-R vs. O-R: p=0.6161<br>E-R vs. S-R: p=0.0837<br>O-R vs. S-R: p=0.4498<br>KO<br>E-R vs. O-R: p=0.8904<br>E-R vs. S-R: p=0.4888<br>O-R vs. S-R: p=0.7729
						O-R: -0.0273±0.01758	O-R: -0.01727 (-0.07068, 0.01938)			
						S-R: -0.01775±0.01553	S-R: -0.01403 (-0.05713, 0.0128)			
					KO neuron n=40 / mice n=5	E-R: -0.05127±0.01432	E-R: -0.04377 (-0.1078, 0.008637)			
						O-R: -0.04783±0.01427	O-R: -0.04633 (-0.1207, 0.001874)			
						S-R: -0.0427±0.01386	S-R: -0.03002 (-0.1215, 0.00204)			
**Supplimentary figure 4a**	Linear Chamber	WT pre,post-Alpha PSD	M	12wks	mice n=5	Empty WT=52	Pre:0.0103±0.000493	Two Way ANOVA with Tukey's test F(1,104)=4.022	time 0.0329<br>target0.021<br>interaction 0.042	pre E vs S p=0.082
							Post:0.00998±0.000493			post E vs S p=0.009
						Social WT=52	Pre:0.0133±0.000493			Empty pre vs post p=0.077
							Post:0.014±0.000493			Social pre vs post p=0.354
						object WT=52	Pre:0.013±0.000493			object pre vs post p=0.018
							Post:0.0112±0.000493			
		KO pre,post-Alpha PSD	M	12wks	mice n=5	Empty KO=53	Pre:0.0133±0.000557	Two Way ANOVA with Tukey's test F(1,102)=14.414	time <0.001<br>target 0.145<br>interaction 0.533	pre E vs S p>0.05
							Post:0.00938±0.000557			post E vs S p>0.05
						Social KO=53	Pre:0.0142±0.000557			Empty pre vs post p=0.004
							Post:0.0113±0.000557			Social pre vs post p=0.003
						object KO=53	Pre:0.0142±0.000557			object pre vs post p<0.001
							Post:0.00908±0.000557			
**Supplimentary figure 4b**	Linear Chamber	WT pre,post-Theta PSD	M	12wks	mice n=5	Empty WT=52	Pre:0.0315±0.00157	Two Way ANOVA with Tukey's test F(1,104)=4.907	time 0.449<br>target0.258<br>interaction 0.326	
							Post:0.0331±0.00157			
						Social WT=52	Pre:0.0341±0.00157			
							Post:0.033±0.00157			
						object WT=52	Pre:0.0369±0.00157			
							Post:0.038±0.00157			
		KO pre,post-Theta PSD	M	12wks	mice n=5	Empty KO=53	Pre:0.0508±0.000151	Two Way ANOVA with Tukey's test F(1,102)=14.22	time 0.001<br>target 0.999<br>interaction 0.233	pre E vs S p>0.05
							Post:0.0046±0.000151			post E vs S p>0.05
						Social KO=53	Pre:0.0517±0.000151			Empty pre vs post p=0.077
							Post:0.0452±0.000151			Social pre vs post p=0.017
						object KO=53	Pre:0.046±0.000151			object pre vs post p<0.001
							Post:0.0436±0.000151			

**Supplimentary figure 4c**	Linear Chamber	WT<br>pre,post-Delta PSD	M	12wks	mice n=5	Empty WT=52<br>Social WT=52<br>object WT=52	Pre:0.399±0.000346<br>Post:0.0393±0.000346<br>Pre:0.0396±0.000346<br>Post:0.386±0.000346<br>Pre:0.0407±0.000346<br>Post:0.397±0.000346	Two Way ANOVA with Tukey's test<br>F(1,104)=4.848	time 0.032<br>target0.016<br>interaction 0.807	pre E vs S p=0.093<br>post E vs S p>0.05<br>Empty pre vs post p=0.077<br>Social pre vs post p=0.072<br>object pre vs post p<0.001
		KO<br>pre,post-Delta PSD	M	12wks	mice n=5	Empty KO=53<br>Social KO=53<br>object KO=53	Pre:0.392±0.000307<br>Post:0.418±0.000307<br>Pre:0.392±0.000307<br>Post:0.409±0.000307<br>Pre:0.403±0.000307<br>Post:0.422±0.000307	Two Way ANOVA with Tukey's test<br>F(1,102)=15.036	time <0.001<br>target 0.014<br>interaction 0.29	pre E vs S p=0.105<br>post E vs S p>0.05<br>Empty pre vs post p=0.077<br>Social pre vs post p=0.01<br>object pre vs post p=0.004
**Supplimentary figure 5**	Immuno<br>-histochemistry	Density of<br>Pv-positive neurons	M	>12wks	WT : 33 slices / 5mice<br>KO : 36 neurons / 5mice	WT Cg1:9.132±0.8355<br>KO Cg1:4.6361.316	7.421<br>(6.655, 9.168)<br>2.908<br>(2.452, 3.557)	Mann Whitney,<br>two-tailed	p=0.0556	ns
						WT PrL:3.7030.7434<br>KO PrL:1.8190.6602	2.03<br>(1.876, 3.824)<br>0.986<br>(0.8916, 1.299)	Mann Whitney,<br>two-tailed	p=0.0556	ns
						WT IL:2.9660.7229<br>KO IL:1.6620.4909	1.607<br>(1.28, 2.329)<br>0.8354<br>(0.7818, 1.504)	Unpaired t test, two-tailed	p=0.1741	ns
						WT Total:5.4970.618<br>KO Total:2.7730.8376	4.156<br>(3.735, 5.526)<br>1.764<br>(1.747, 1.796)	Mann Whitney,<br>two-tailed	p=0.0556	ns
		Density of<br>Som-positive neurons	M	>12wks	WT : 33 slices / 5mice<br>KO : 36 neurons / 5mice	WT Cg144.65±2.301<br>KO Cg137.421.446	39.47<br>(37.54, 46.03)<br>34.23<br>(34.14, 37.39)	Unpaired t test, two-tailed	p=0.0287	*
						WT PrL33.843.295<br>KO PrL322.921	27.05<br>(22.3, 34.86)<br>26.92<br>(24.41, 30.86)	Unpaired t test, two-tailed	p=0.6875	ns
						WT IL34.293.809<br>KO IL35.977.864	27.72<br>(23.5, 32.68)<br>22.44<br>(5.665, 40.78)	Unpaired t test, two-tailed	p=0.8517	ns
						WT Total37.592.503<br>KO Total34.482.848	32.98<br>(28.64, 37.39)<br>29.41<br>(25.57, 34.65)	Unpaired t test, two-tailed	p=0.4357	ns
**Supplimentary Figure 6a**	Slice electrophysiology with optogenetic stimulation	10Hz optoSTIM (V-clamp)<br>Stimulated PV	M	12wks	WT (firing) : 11 neurons / 3 mice<br>KO (firing) : 11 neurons / 3 mice	WT : -2017 ± 528.3<br>KO : -2216 ± 385.1	WT : -2619 (-3892, -228.9)<br>KO : -2425 (-3817, -957.1)	Mann Whitney test	p-0.6994	ns
		10Hz optoSTIM (V-clamp)<br>Stimulated PV	M	12wks	WT (no fire) : 11 neurons / 3 mice<br>KO (no fire) : 11 neurons / 3 mice	WT : -605.6 ± 179.8<br>KO : -704.7 ± 94.75	WT : -354 (-1068, -190.1)<br>KO : -651 (-825.1, -316.1)	Unpaired t test	p=0.6310	ns
**Supplimentary figure 6b**	Slice electrophysiology with optogenetic stimulation	10Hz optoSTIM<br>Neighboring PV<br>Ratio diagram	M	12wks	WT (ACSF) : 504 epochs/ 10mice<br>WT (Synaptic blockers) : 96 epochs/ 3mice	Chi-square, df	WT (ACSF) : 151 (332, 21)<br>WT (Synaptic blockers) : 26 (69, 1)	Chi-square	p=0.2455	ns
		10Hz optoSTIM<br>Neighboring PV<br>Ratio diagram	M	12wks	KO (ACSF) : 456 epochs/ 15mice<br>KO (Synaptic blockers) : 96 epochs/ 3mice	Chi-square, df	KO (ACSF) : 92 (111, 61)<br>KO (Synaptic blockers) : 12 (72, 12)	Chi-square	p<0.0001	****
		10Hz optoSTIM<br>Neighboring PV<br>Firing rate	M	12wks	WT (ACSF) : 47 neurons / 10 mice<br>KO (ACSF) : 42 neurons / 15 mice<br>WT (synaptic blockers) : 8 neurons / 3 mice<br>KO (synaptic blockers) :8 neurons / 3 mice	WT (ACSF) : 4.202 ± 0.901<br>WT (synaptic blockers) : 3.978 ± 1.943<br>KO (ACSF) : 11.984 ± 1.491<br>KO (synaptic blockers) : 11.119 ± 1.789	WT (ACSF) : 0 (10, 0)<br>WT (synaptic blockers) : 0 (10.677, 0)<br>KO (ACSF) : 10 (16.875, 6.667)<br>KO (synaptic blockers) : 10.899 (14.296, 10.885)	Two-way repeated measures of ANOVA<br>with Šídák's multiple comparisons test	Interaction F (1, 101) = 0.02347P=0.8785<br>Drug F (1, 101) = 0.06795P=0.7949<br>Group F (1, 101) = 12.75***P=0.0005	ACSF - synaptic blockers<br>WT, ns (p=0.9963)<br>KO, ns (p=0.9477)
**Supplimentary figure 8**	Slice electrophysiology with optogenetic stimulation	10Hz optoSTIM<br>Neighboring PV<br>Ratio diagram	M	12wks	WT (10, 40, 80 Hz) : 216 epochs / 4mice	Chi-square, df	WT (10 Hz) : 91 (119, 6)<br>WT (40 Hz) : 143 (719, 2)<br>WT (80 Hz) : 164 (698, 2)	Chi-square	WT 10 vs. 40 Hz : p<0.0001<br>WT 10 vs. 80 Hz : p<0.0001	WT 10 vs. 40 Hz : ****<br>WT 10 vs. 80 Hz : ****
		10Hz optoSTIM<br>Neighboring PV<br>Ratio diagram	M	12wks	KO (10, 40, 80 Hz) : 252 epochs / 4mice	Chi-square, df	KO (10 Hz) : 58 (155, 39)<br>KO (40 Hz) : 469 (537, 2)<br>KO (80 Hz) : 471 (528, 9)	Chi-square	KO 10 vs. 40 Hz : p<0.0001<br>KO 10 vs. 80 Hz : p<0.0001	KO 10 vs. 40 Hz : ****<br>KO 10 vs. 80 Hz : ****
		10Hz optoSTIM<br>Neighboring PV<br>Firing rate	M	12wks	WT (10, 40, 80 Hz) : 18 neurons / 4 mice<br>KO (10, 40, 80 Hz) : 21 neurons / 4 mice	WT 10 Hz : 4.990 ± 1.373<br>WT 40 Hz : 10.28 ± 3.260<br>WT 80 Hz : 18.09 ± 5.918<br>KO 10 Hz : 10.65 ± 1.450<br>KO 40 Hz : 28.75 ± 3.357<br>KO 80 Hz : 55.16 ± 6.501	WT 10 Hz : 0 (0, 10.89)<br>WT 40 Hz : 0 (0, 18.91)<br>WT 80 Hz : 6.817 (0, 25.98)<br>KO 10 Hz : 10.89 (8.764, 13.16)<br>KO 40 Hz : 34.46 (19.30, 40.42)<br>KO 80 Hz : 58.37 (30.83, 80.75)	Two-way ANOVA<br>with Tukey's multiple comparisons test	WT, Hz, F (2, 34) = 5.320P=0.0098<br>KO, Hz, F (2, 40) = 50.12P<0.0001	WT :<br>10 - 40 Hz, ns (p=0.3996)<br>10 - 80 Hz, ** (p=0.0073)<br>KO :<br>10 - 40 Hz, *** (p=0.0007)<br>10 - 80 Hz, **** (p<0.0001)
**Supplimentary figure 10**	Sholl analysis	Intersections	M	10wks	WT: 4 neurons / 3 mice	0 µm: 4.25±0.75<br>10 µm: 6.5±1.708<br>20 µm: 9.75±1.702<br>30 µm: 13±2.677<br>40 µm: 14.5±3.122<br>50 µm: 15.75±3.705<br>60 µm: 15.75±3.683<br>70 µm: 15.5±3.884<br>80 µm: 14.25±3.705<br>90 µm: 14±3.582<br>100 µm: 15.25±4.479<br>110 µm: 14±3.24<br>120 µm: 13±2.799<br>130 µm: 12±2.38<br>140 µm: 11±1.683<br>150 µm: 10.75±2.869<br>160 µm: 8.75±2.056<br>170 µm: 8±1.472<br>180 µm: 6.75±1.887<br>190 µm: 6.25±1.652<br>200 µm: 5.5±1.258<br>210 µm: 4.75±0.9465<br>220 µm: 4±0.8165<br>230 µm: 3.75±0.8539<br>240 µm: 2.5±0.6455<br>250 µm: 2±0.8165<br>260 µm: 1.75±0.8539<br>270 µm: 1.25±0.75<br>280 µm: 1.25±0.75<br>290 µm: 1±0.5774<br>300 µm: 1±0.5774<br>310 µm: 1±0.5774<br>320 µm: 0.75±0.4787<br>330 µm: 0.5±0.5<br>340 µm: 0.25±0.25<br>350 µm: 0±0<br>360 µm: 0±0<br>370 µm: 0±0<br>380 µm: 0±0<br>390 µm: 0±0<br>400 µm: 0±0	0 µm: 5 (2.75, 5)<br>10 µm: 7 (3, 9.5)<br>20 µm: 10.5 (6.25, 12.5)<br>30 µm: 15.5 (7.5, 16)<br>40 µm: 15.5 (8.25, 19.75)<br>50 µm: 16.5 (8.5, 22.25)<br>60 µm: 15.5 (9, 22.75)<br>70 µm: 15.5 (8.25, 22.75)<br>80 µm: 13.5 (7.75, 21.5)<br>90 µm: 12.5 (8.25, 21.25)<br>100 µm: 13 (8.5, 24.25)<br>110 µm: 12.5 (8.75, 20.75)<br>120 µm: 11.5 (8.75, 18.75)<br>130 µm: 10 (9, 17)<br>140 µm: 9.5 (9, 14.5)<br>150 µm: 9 (6.5, 16.75)<br>160 µm: 8 (5.25, 13)<br>170 µm: 8 (5.25, 10.75)<br>180 µm: 7 (3, 10.25)<br>190 µm: 6.5 (3, 9.25)<br>200 µm: 6 (3, 7.5)<br>210 µm: 5.5 (2.75, 6)<br>220 µm: 4 (2.5, 5.5)<br>230 µm: 3.5 (2.25, 5.5)<br>240 µm: 2.5 (1.25, 3.75)<br>250 µm: 2 (0.5, 3.5)<br>260 µm: 1.5 (0.25, 3.5)<br>270 µm: 1 (0, 2.75)<br>280 µm: 1 (0, 2.75)<br>290 µm: 1 (0, 2)<br>300 µm: 1 (0, 2)<br>310 µm: 1 (0, 2)<br>320 µm: 0.5 (0, 1.75)<br>330 µm: 0 (0, 1.5)<br>340 µm: 0 (0, 0.75)<br>350 µm: 0 (0, 0)<br>360 µm: 0 (0, 0)<br>370 µm: 0 (0, 0)<br>380 µm: 0 (0, 0)<br>390 µm: 0 (0, 0)<br>400 µm: 0 (0, 0)	Repeated measures of Two Way ANOVA	Interaction F (40, 200) = 0.1183, P>0.9999;<br>Genotype F (1, 5) = 0.02754, P=0.8747;<br>Distance F (40, 200) = 26.29, P<0.0001	
					KO: 3 neurons / 3 mice	0 µm: 5.667±0.8819<br>10 µm: 8±1<br>20 µm: 11±1.528<br>30 µm: 12.67±2.028<br>40 µm: 15±1.528<br>50 µm: 14.67±0.6667<br>60 µm: 15.67±0.6667<br>70 µm: 15±0<br>80 µm: 15.67±0.3333<br>90 µm: 14±1.528<br>100 µm: 13.67±1.667<br>110 µm: 14.67±2.186<br>120 µm: 12.33±0.8819<br>130 µm: 12±0<br>140 µm: 12.33±0.3333<br>150 µm: 9.333±1.453<br>160 µm: 8.667±1.202<br>170 µm: 8±1.528<br>180 µm: 6.333±1.333<br>190 µm: 5.667±1.202<br>200 µm: 4.333±1.856<br>210 µm: 4±2<br>220 µm: 3±2<br>230 µm: 2±1.528<br>240 µm: 2±1.528<br>250 µm: 1.667±1.202<br>260 µm: 1±0.5774<br>270 µm: 1±0.5774<br>280 µm: 0.6667±0.3333<br>290 µm: 0.6667±0.3333<br>300 µm: 0.3333±0.3333<br>310 µm: 0.3333±0.3333<br>320 µm: 0.3333±0.3333<br>330 µm: 0±0<br>340 µm: 0±0<br>350 µm: 0±0<br>360 µm: 0±0<br>370 µm: 0±0<br>380 µm: 0±0<br>390 µm: 0±0<br>400 µm: 0±0	0 µm: 6 (4, 7)<br>10 µm: 7 (7, 10)<br>20 µm: 12 (8, 13)<br>30 µm: 13 (9, 16)<br>40 µm: 16 (12, 17)<br>50 µm: 14 (14, 16)<br>60 µm: 15 (15, 17)<br>70 µm: 15 (15, 15)<br>80 µm: 16 (15, 16)<br>90 µm: 13 (12, 17)<br>100 µm: 12 (12, 17)<br>110 µm: 13 (12, 19)<br>120 µm: 12 (11, 14)<br>130 µm: 12 (12, 12)<br>140 µm: 12 (12, 13)<br>150 µm: 9 (7, 12)<br>160 µm: 8 (7, 11)<br>170 µm: 7 (6, 11)<br>180 µm: 5 (5, 9)<br>190 µm: 5 (4, 8)<br>200 µm: 3 (2, 8)<br>210 µm: 2 (2, 8)<br>220 µm: 1 (1, 7)<br>230 µm: 1 (0, 5)<br>240 µm: 1 (0, 5)<br>250 µm: 1 (0, 4)<br>260 µm: 1 (0, 2)<br>270 µm: 1 (0, 2)<br>280 µm: 1 (0, 1)<br>290 µm: 1 (0, 1)<br>300 µm: 0 (0, 1)<br>310 µm: 0 (0, 1)<br>320 µm: 0 (0, 1)<br>330 µm: 0 (0, 0)<br>340 µm: 0 (0, 0)<br>350 µm: 0 (0, 0)<br>360 µm: 0 (0, 0)<br>370 µm: 0 (0, 0)<br>380 µm: 0 (0, 0)<br>390 µm: 0 (0, 0)<br>400 µm: 0 (0, 0)			
**Supplimentary figure 11a**	Slice electrophysiology with optogenetic stimulation	10Hz optoSTIM<br>Neighboring PV<br>Ratio diagram	M	12wks	WT : 240 epochs / 4mice<br>WT+DCS : 240 epochs/ 4mice	Chi-square, df	WT : 62 (154, 24)<br>WT+DCS : 67 (135, 38)	Chi-square	p=0.1000	ns
		10Hz optoSTIM<br>Neighboring PV<br>Ratio diagram	M	12wks	KO : 192 epochs / 4mice<br>KO+DCS : 192 epochs/ 4mice	Chi-square, df	KO : 53 (139, 1)<br>KO+DCS : 52 (140, 1)	Chi-square	p=0.9935	ns
		10Hz optoSTIM<br>Neighboring PV<br>Firing rate	M	12wks	WT : 20 neurons / 4 mice<br>WT + DCS : 20 neurons / 4 mice<br>KO : 16 neurons / 4 mice<br>KO + DCS : 16 neurons / 4 mice	WT : 22.571 ± 3.229<br>WT+DCS : 26.154 ± 3.903<br>KO : 25.165 ± 3.850<br>KO+DCS: 25.925 ± 3.565	WT : 21.654 (29.923, 12.025)<br>WT+DCS : 21.637 (39.759, 11.190)<br>KO : 19.697 (31.005, 15.198)<br>KO+DCS: 22.109 (34.384, 14.978)	Two-way repeated measures of ANOVA<br>with Šídák's multiple comparisons test	Interaction F (1, 34) = 1.002, P=0.3238<br>Treatment F (1, 34) = 2.372, P=0.1328<br>genotype F (1, 34) = 0.05572, P=0.8148	ns
**Supplimentary figure 12**	Three chamber test with optogenetic stimulation	WT social	M	12wks	WT_S	same as figure 6c-f		Repeated two way ANOVA test<br>with Sidak's multiple comparison	interaction F(6,104)=1.226 p=0.2991<br>lightF(1.972, 102.5)=0.0248<br>p=0.9742<br>opsin F(3,52)=2.008, p=0.1242	
		KO social			KO_S			Repeated two way ANOVA test<br>with Sidak's multiple comparison	interaction F(6,94)=2.372, p=0.0354<br>lightF(1.913, 89.89)=1.716<br>p=0.1868<br>opsin F(3,52)=2.515, p=0.0697	
		WT object			WT_O			Repeated two way ANOVA test<br>with Sidak's multiple comparison	interaction.F(6,106)=4.547, p=0.0004<br>light F(1.534, 81.28) p=0.0455<br>opsin F(3,53)=0.8957, p=0.4495	
		KO object			KO_O			Repeated two way ANOVA test<br>with Sidak's multiple comparison	interaction F(6,96)=1.745, p=0.1189<br>lightF(1.999, 95.97)=5.071 p=0.0081<br>opsin F(3,48)=2.515, p=0.4332	

Supplimentary figure 13a-d	Open field test with optogenetic stiumulation	Distant moved-WT	12-19wks	M	WT EYFP n=12<br>ChR2n=16	1.off	EYFP: 1497±158.6<br>ChR2: 17861497±131.0	Repeated measures of Two Way ANOVA with Bonferroni's test F(1,104)=23.40	time <0.0001<br>virus 0.4969<br>interaction 0.0003<br>matching <0.0001	
						2.on	EYFP:1091±157.1<br>ChR2:1342±122.3			
						3.off	EYFP:1097±122.3<br>ChR2:1095±96.49			
						4.on	EYFP:975.2±167.5<br>ChR2:1243±99.75			
						5.off	EYFP:1203±119.0<br>ChR2:939.8±103.0			
		Distant moved-KO			KO EYFP n=12<br>ChR2n=9	1.off	EYFP:3434±241.7<br>ChR2:3307±204.8	Repeated measures of Two Way ANOVA with Bonferroni's test F(1,76)=3.751	time 0.0077<br>virus 0.4748<br>interaction 0.7917<br>matching <0.0001	
						2.on	EYFP:3369±346.3<br>ChR2:2803±277.4			
						3.off	EYFP:3401±475.1<br>ChR2:2876±464.4			
						4.on	EYFP:3262±490.1<br>ChR2:2774±440.4			
						5.off	EYFP:2809±590.3<br>ChR2:2465±450.3			
		Cneter time-WT			WT EYFP n=12<br>ChR2n=16	1.off	EYFP:8.896±1.703<br>ChR2:9.633±2.510	Repeated measures of Two Way ANOVA with Bonferroni's test F(1,96)=1.007	time 0.4078<br>virus 0.8759<br>interaction 0.6356<br>matching <0.0001	
						2.on	EYFP:9.455±2.258<br>ChR27.646±2.449			
						3.off	EYFP:9.455±3.660<br>ChR2:7.646±2.449			
						4.on	EYFP:9.735±3.660<br>ChR2:6.514±1.451			
						5.off	EYFP:6.206±2.140<br>ChR2:7.222±2.419			
		Center time-KO			KO EYFP n=12<br>ChR2n=9	1.off	EYFP:18.76±2.544<br>ChR2:14.87±14.34	Repeated measures of Two Way ANOVA with Bonferroni's test F(1,104)=0.9485	time 0.4405<br>virus 0.9983<br>interaction 0.7003<br>matching <0.0001	
						2.on	EYFP:14.34±2.539<br>ChR2:13.02±5.317			
						3.off	EYFP:11.81±3.141<br>ChR2:12.30±2.388			
						4.on	EYFP:11.65±2.568<br>ChR2:15.02±6.093			
						5.off	EYFP: 14.29±4.973<br>ChR2:15.58±6.316			
Supplimentary figure 13e	Three chamber test with optogenetic stimulation	Shank2 WT PV-cre;<br>AAV-ChR2-WT	M	>12wks	n=9	Social:120.9±17.14	Social:120.9<br>(80.37, 161.4)	Unpaired t test, two-tailed	p=0.0395	*
						Object:73.46±11.96	Object:73.46<br>(45.18, 101.7)			
		Shank2 KO PV-cre;<br>AAV-ChR2-KO			n=6	Social:129.2±13.68	Social:129.2<br>(100.1, 158.2)	Unpaired t test, two-tailed	p=0.0975	ns
						Object:90.11±15.92	Object:90.11<br>(56.29, 123.9)			
Supplimentary figure 14b	in vivo recording with optogenetic stimulation	PV-cre;<br>AAV-ChETA	M	>12wks	n=28	off: 2.959±0.4560	2.2 (1.123, 4.711)	Wilcoxon matched-pairs signed rank test, two-tailed	p=0.4117	ns
						on: 3.020±0.4598	2.441 (1.179, 4.138)			
Supplimentary figure 14c	in vivo recording with optogenetic stimulation	PV-cre;<br>AAV-SSFO	M	>12wks	n=21	off: 5.345±1.642	3.006 (1.836, 6.031)	Wilcoxon matched-pairs signed rank test, two-tailed	p=0.0239	*
						on: 4.201±1.126	2.227 (1.373, 4.453)			
Supplimentary figure 15c	in vivo recording with optogenetic stimulation	Shank2 WT PV-cre;<br>AAV-EYFP	M	>12wks	n=194 /7			Chi-square test	p=0.915	ns
		Shank2 WT PV-cre;<br>AAV-ChR2			n=72 /8					
Supplimentary figure 16a	Western blotting	Connexin-36/actin<br>(normalized to control value)	HEK293T		n=3	Control: 1±0.1285	Control: 1.005<br>(0.7748, 1.22)	Ordinary one-way ANOVA	0.0868	
					n=3	shRNA1: 0.6431±0.04486	shRNA1: 0.6144<br>(0.5839, 0.7311)			
Supplimentary figure 16b	Openfield	shRNA1<br>Distance moved	M	>12wks	Control: n=9<br>Knockdown: n=9	Control: 148.1±6.33	Control: 139.9<br>(131.5, 169.8)	Unpaired t test,<br>two-tailed	0.7955	ns
						KD: 151.2±9.975	KD: 149.7<br>(123.5, 173.7)			
	Openfield	shRNA1<br>Center time				Control: 217.5±20.9	Control: 223.2<br>(177, 265)	Unpaired t test,<br>two-tailed	0.6243	ns
						KD: 237.4±33.94	KD: 218<br>(159.4, 322.8)			
	Openfield	shRNA2<br>Distance moved	M	>10wks	Control: n=10<br>Knockdown: n=10	Control: 143.1±7.337	Control: 143.4<br>(127.8, 163.5)	Unpaired t test,<br>two-tailed	0.7753	ns
						KD: 146.4±8.875	KD: 149.4<br>(122.4, 162)			
	Openfield	shRNA2<br>Center time				Control: 249.3±31.42	Control: 236.3<br>(169.2, 329.1)	Unpaired t test,<br>two-tailed	0.2376	ns
						KD: 204.7±18.67	KD: 199.2<br>(178.3, 223.7)			

Supplementary Table 2. Intrinsic properties of pyramidal neurons and Pv neurons in WT and Shank2-KO neurons.

Comparable intrinsic properties in WT and *Shank2*$^{-/-}$ pyramidal and Pv neurons in the mPFC (> 12 weeks). Pv neurons were marked by ChR2-EYFP expression. IR, input resistance; AP, action potential, FWHM, full width at half maximum, AHP, after hyperpolarization, ISI, inter-spike interval. Data: mean ± SEM. (n = 21 neurons from 5 mice [WT-PN/pyramidal neurons], 13/5 [KO-PN], 24/6 [WT-Pv], and 16/4 [KO-Pv], ns, not significant, Student's t-test).

							Stat method	
Property	WT Py (n=21)	KO Py (n=13)	Statistics	WT Pv (n=24)	KO Pv (n=19)	Statistics	Py (WT vs. KO)	PV (WT vs. KO)
IR (passive) (MΩ)	75.23±5.914	73.90±8.765	p=0.9165	74.87±2.452	78.50±1.865	p=0.5012	Mann-Whitney test	Mann-Whitney test
Sag (mV)	20.44±0.9932	21.69±1.626	p=0.4894	24.44±1.168	26.06±0.9098	p=0.0893	Unpaired t-test	Mann-Whitney test
AP threshold (mV)	-42.73±1.770	-38.70±1.535	p=0.2608	-38.73±1.083	-39.24±2.107	p=0.6240	Mann-Whitney test	Mann-Whitney test
AP amplitude (mV)	69.71±1.604	71.30±3.112	p=0.5817	47.97±1.639	48.50±1.629	p=0.8245	Mann-Whitney test	Unpaired t-test
FWHM (ms)	1.309±0.05330	1.445±0.1185	p=0.2431	0.7982±0.02326	0.8608±0.07486	p=0.9133	Unpaired t-test	Mann-Whitney test
AHP (mV)[a]	12.28±0.8922	10.21±1.354	p=0.3955	15.86±0.9857	17.13±1.484	p=0.6896	Mann-Whitney test	Mann-Whitney test
Rheobase (pA)	135.7±10.99	150.0±30.56	p=0.6160	239.6±15.92	181.6±17.61	p=0.0616	Mann-Whitney test	Unpaired t-test
ISI ratio	4.225±0.3090	3.746±0.5036	p=0.3963	1.307±0.02791	1.933±0.4037	p=0.3255	Unpaired t-test	Mann-Whitney test

References

1. Lee, E.*, et al.* Enhanced Neuronal Activity in the Medial Prefrontal Cortex during Social Approach Behavior. *J Neurosci* **36**, 6926-6936 (2016).
2. Latimer, K.W., Yates, J.L., Meister, M.L., Huk, A.C. & Pillow, J.W. NEURONAL MODELING. Single-trial spike trains in parietal cortex reveal discrete steps during decision-making. *Science* **349**, 184-187 (2015).
3. Rudy, B., Fishell, G., Lee, S. & Hjerling-Leffler, J. Three groups of interneurons account for nearly 100% of neocortical GABAergic neurons. *Dev Neurobiol* **71**, 45-61 (2011).
