# Supplementary material for: Excitatory synapses and gap junctions cooperate to improve Pv neuronal burst firing and cortical social cognition in Shank2-mutant mice
Source: Nat Commun. 2021 Aug 25;12:5116. doi: 10.1038/s41467-021-25356-2 (PMC8387434; doi:10.1038/s41467-021-25356-2)
Supplement: Supplementary file 3 — Reporting Summary [file 41467_2021_25356_MOESM3_ESM.pdf]

nature portfolio

Corresponding author(s): Eunjoon Kim

Last updated by author(s): 2021-7-19

Reporting Summary

Nature Portfolio wishes to improve the reproducibility of the work that we publish. This form provides structure for consistency and transparency in reporting. For further information on Nature Portfolio policies, see our Editorial Policies and the Editorial Policy Checklist.

Statistics

For all statistical analyses, confirm that the following items are present in the figure legend, table legend, main text, or Methods section.

n/a	Confirmed	
☐	☒	The exact sample size (n) for each experimental group/condition, given as a discrete number and unit of measurement
☐	☒	A statement on whether measurements were taken from distinct samples or whether the same sample was measured repeatedly
☐	☒	The statistical test(s) used AND whether they are one- or two-sided<br>*Only common tests should be described solely by name; describe more complex techniques in the Methods section.*
☒	☐	A description of all covariates tested
☐	☒	A description of any assumptions or corrections, such as tests of normality and adjustment for multiple comparisons
☐	☒	A full description of the statistical parameters including central tendency (e.g. means) or other basic estimates (e.g. regression coefficient) AND variation (e.g. standard deviation) or associated estimates of uncertainty (e.g. confidence intervals)
☐	☒	For null hypothesis testing, the test statistic (e.g. F, t, r) with confidence intervals, effect sizes, degrees of freedom and P value noted<br>*Give P values as exact values whenever suitable.*
☒	☐	For Bayesian analysis, information on the choice of priors and Markov chain Monte Carlo settings
☒	☐	For hierarchical and complex designs, identification of the appropriate level for tests and full reporting of outcomes
☒	☐	Estimates of effect sizes (e.g. Cohen's d, Pearson's r), indicating how they were calculated

Our web collection on statistics for biologists contains articles on many of the points above.

Software and code

Policy information about availability of computer code

Data collection	Clampex 10.7 and pCLAMP 10.7 were used to acquire in vitro electrophysiological data, and Cheetah5 was used to acquire in vivo neuronal activities.
Data analysis	All behavioral data were analyzed using Ethovision13 (Noduls). ImageJ program was used to analyze immunohistochemistry data. Prism9.1.2 (GraphPad) was used for statistical analyses. Results with large n numbers were analyzed using a custom code (Matlab2020b). Single-unit data from in vivo tetrode recordings were extracted using MClust 4.0. The code for the custom-made miniature event analysis program is available at https://github.com/parkgilbong/Minhee_Analysis_Pack.

For manuscripts utilizing custom algorithms or software that are central to the research but not yet described in published literature, software must be made available to editors and reviewers. We strongly encourage code deposition in a community repository (e.g. GitHub). See the Nature Portfolio guidelines for submitting code & software for further information.

Data

Policy information about availability of data

All manuscripts must include a data availability statement. This statement should provide the following information, where applicable:

- Accession codes, unique identifiers, or web links for publicly available datasets
- A description of any restrictions on data availability
- For clinical datasets or third party data, please ensure that the statement adheres to our policy

The custom code used in this manuscript will be available upon e-mail request.

Field-specific reporting

Please select the one below that is the best fit for your research. If you are not sure, read the appropriate sections before making your selection.

- [x] Life sciences
- [ ] Behavioural & social sciences
- [ ] Ecological, evolutionary & environmental sciences

For a reference copy of the document with all sections, see nature.com/documents/nr-reporting-summary-flat.pdf

Life sciences study design

All studies must disclose on these points even when the disclosure is negative.

Sample size	Sample sizes were chosen based on our previous studies using the same Shank2-mutant mice (Won et al., Nature 486:261, 2012; Chung et al., Biol Psych 85:534, 2019).
Data exclusions	Outliers were excluded using the Grubbs' test (also known as Extreme Studentized Deviate (ESD) test) and the cut off at $p < 0.05$. Exclusion criteria for in vivo data are described in Methods. Briefly, neurons with mean-firing rates smaller than 0.5 Hz were excluded for the possibility of contamination with noise. For two-way ANOVA test to determine target specificity/discrimination of recorded neurons, the experiments that which do not contain all six trials for E-E, 1st S-O and 2nd S-O sessions were excluded for difficulty of running statistical tests.
Replication	All experiments were replicated through multiple cohort/mice analysis, where applicable. All replication attempts were successful.
Randomization	For randominization, we counterbalanced vehicle-drug treatments and left-right target positions.
Blinding	All experiments and analyses were performed by investigators who are blind to the genotype or group allocations of the mice.

Reporting for specific materials, systems and methods

We require information from authors about some types of materials, experimental systems and methods used in many studies. Here, indicate whether each material, system or method listed is relevant to your study. If you are not sure if a list item applies to your research, read the appropriate section before selecting a response.

Materials & experimental systems

n/a	Involved in the study	
☐	☒	Antibodies
☒	☐	Eukaryotic cell lines
☒	☐	Palaeontology and archaeology
☐	☒	Animals and other organisms
☒	☐	Human research participants
☒	☐	Clinical data
☒	☐	Dual use research of concern

Methods

n/a	Involved in the study	
☒	☐	ChIP-seq
☒	☐	Flow cytometry
☒	☐	MRI-based neuroimaging

Antibodies

Antibodies used	Mouse monoclonal parvalbumin antibody (Milipore, MAB1572), Rabbit polyclonal calretinin antibody (Millipore, AB5054), Rabbit polyclonal somatostatin antibody (Peninsula, T-4547), Mouse HA monoclonal antibody(MBL M180-3).
Validation	Websites for antibody validations:<br>Mouse monoclonal parvalbumin antibody (Milipore, MAB1572):https://www.merckmillipore.com/KR/ko/product/Anti-Parvalbumin-Antibody,MM_NF-MAB1572<br>Rabbit polyclonal calretinin antibody (Millipore, AB5054) and Rabbit polyclonal somatostatin antibody (Peninsula, T-4547):http://www.bma.ch/en/products/t-4547<br>Rabbit polyclonal somatostatin antibody (Peninsula, T-4545): http://www.bma.ch/en/products/t-4545<br>Mouse HA monoclonal antibody (MBL M180-3): https://www.biocompare.com/9776-Antibodies/2304439-antiHAtag/

Animals and other organisms

Policy information about studies involving animals; ARRIVE guidelines recommended for reporting animal research

Laboratory animals	Mice, C57B/6N for Shank2 mice and C57B/6J for PV-cre mice. Adult male mice at the age of 10-16 weeks.<br>experiments. All animals were fed ad libitum and housed under 12 h light/dark cycle (light phase from 1 am to 1 pm) under 21 degree celcius and 50-60% humidity.
Wild animals	No wild animals were used in this study.

Field-collected samples	No field-collected samples were used.
Ethics oversight	Mouse maintenance and breeding followed the guideline of the Animal Research Requirements of Korea Advanced Institute of Science and Technology (KAIST) under the approval number of KA2016-30.

Note that full information on the approval of the study protocol must also be provided in the manuscript.
