# Supplementary figures and images for: Excitatory synapses and gap junctions cooperate to improve Pv neuronal burst firing and cortical social cognition in Shank2-mutant mice
Source: Nat Commun. 2021 Aug 25;12:5116. doi: 10.1038/s41467-021-25356-2 (PMC8387434; doi:10.1038/s41467-021-25356-2)

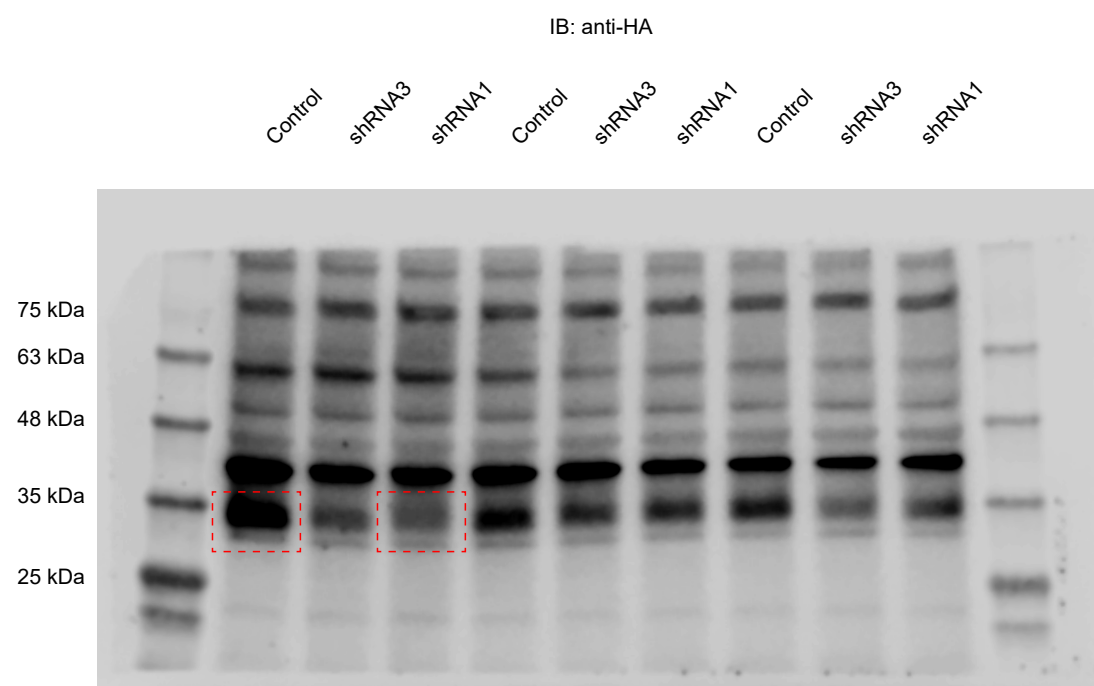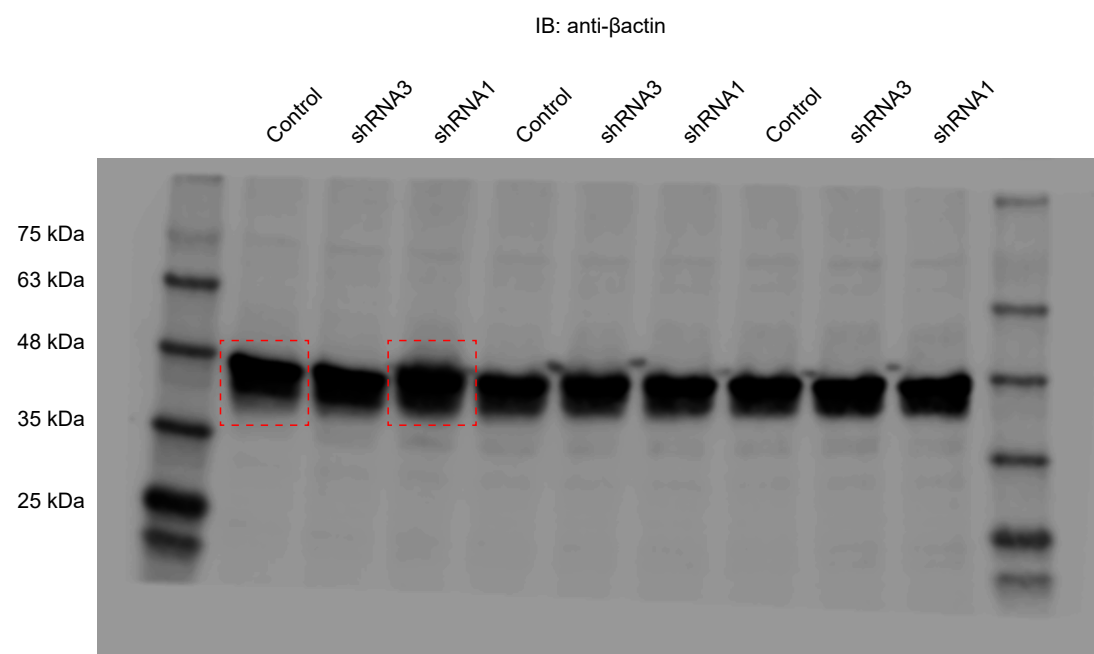

Supplement: Supplementary file 4 — Source data [file 41467_2021_25356_MOESM4_ESM.zip › Source data_immuno blot Lee et al.pdf]
